# Hyaluronidase‐Responsive Bactericidal Cryogel for Promoting Healing of Infected Wounds: Inflammatory Attenuation, ROS Scavenging, and Immune Regulation

**DOI:** 10.1002/advs.202306602

**Published:** 2024-02-13

**Authors:** Menglong Liu, Rui Ding, Zheng Li, Na Xu, Yali Gong, Yong Huang, Jiezhi Jia, Haiyan Du, Yunlong Yu, Gaoxing Luo

**Affiliations:** ^1^ Institute of Burn Research State Key Laboratory of Trauma Burns and Combined Injury Southwest Hospital Third Military Medical University (Army Medical University) Gaotanyan Street, Shapingba District Chongqing 400038 China; ^2^ College of Chemical Engineering and Technology Taiyuan University of Technology Yingze West Street 79 Taiyuan 030024 China

**Keywords:** anti‐bacteria, antimicrobial peptide, cryogel, macrophage regulation, MDR bacteria‐infected wounds

## Abstract

Wounds infected with multidrug‐resistant (MDR) bacteria are increasingly threatening public health and challenging clinical treatments because of intensive bacterial colonization, excessive inflammatory responses, and superabundant oxidative stress. To overcome this malignant burden and promote wound healing, a multifunctional cryogel (HA/TA2/KR2) composed of hyaluronic acid (HA), tannic acid (TA), and KR‐12 peptides is designed. The cryogel exhibited excellent shape‐memory properties, strong absorption performance, and hemostatic capacity. In vitro experiments demonstrated that KR‐12 in the cryogel can be responsively released by stimulation with hyaluronidase produced by bacteria, reaching robust antibacterial activity against *Escherichia coli* (*E. coli)*, MDR *Pseudomonas aeruginosa* (MDR‐PA), and methicillin‐resistant *Staphylococcus aureus* (MRSA) by disrupting bacterial cell membranes. Furthermore, the synergetic effect of KR‐12 and TA can efficiently scavenge ROS and decrease expression of pro‐inflammatory cytokines (tumor necrosis factor (TNF)‐α & interleukin (IL)−6), as well as modulate the macrophage phenotype toward the M2 type. In vivo animal tests indicated that the cryogel can effectively destroy bacteria in the wound and promote healing process via accelerating angiogenesis and re‐epithelialization. Proteomic analysis revealed the underlying mechanism by which the cryogel mainly reshaped the infected wound microenvironment by inhibiting the Nuclear factor kappa B (NF‐κB) signaling pathway and activating the Janus kinase‐Signal transducer and activator of transcription (JAK‐STAT6) signaling pathway. Therefore, the HA/TA2/KR2 cryogel is a promising dressing candidate for MDR bacteria‐infected wound healing.

## Graphical abstract

Wounds infected with multidrug‐resistant (MDR) bacteria are increasingly threatening public health and challenging clinical treatments, needing urgent anti‐infection dressings. The multifunctional HA/TA2/KR2 cryogel composed of hyaluronic acid (HA), tannic acid (TA), and KR‐12 peptides can effectively kill MDR bacteria, inhibit over‐oxidation and manipulate the polarization of macrophages, which can significantly promote angiogenesis and re‐epithelization, finally accelerates infected wound healing.

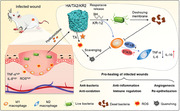

## 1 Introduction

The skin is the largest organ and the most important natural barrier of the human body. It protects internal organs, maintains the balance of body fluids, and prevents the invasion of external harmful substances and bacteria. However, the increasing infection of multidrug‐resistant (MDR) bacteria in skin wounds, such as methicillin‐resistant *Staphylococcus aureus* (MRSA) and MDR *Pseudomonas aeruginosa* (MDR‐PA), has become one of the biggest threats to global public health because of the improper use of antibiotics in the past few decades.^[^ ^1^ ^]^ A recent study predicted that 10 million people will die every year by 2050 because of MDR bacterial infections.^[^ ^2^ ^]^ The World Health Organization (WHO) has declared that current antibacterial therapies are inadequate to meet this challenge and alternatives to antibiotics are urgently needed.^[^ ^3^ ^]^ Wounds infected by MDR bacteria usually experience excessive inflammatory response and oxidative stress, which significantly impairs wound re‐epithelialization and angiogenesis.^[^ ^4^ ^]^ In addition, severely deteriorating wound microenvironment induces the degradation of extracellular matrix (ECM) to provide a breeding ground for bacterial growth, in turn forming a vicious cycle that makes wound healing difficult.^[^ ^5^, ^6^ ^]^ Therefore, killing MDR bacteria, the prerequisite for infected wound healing, has attracted attention worldwide and has been proved to be the effective pathway.

Although there are many highly effective antibacterial strategies for MDR bacteria‐infected wound healing,^[^ ^7^ ^]^ few reports have focused on the post‐antimicrobial stages involving macrophages. Macrophages as the core immune cells in wound tissues exhibit M1 type pro‐inflammatory and M2 type anti‐inflammatory phenotypes. M1 type mainly expresses inflammatory cytokines such as tumor necrosis factor (TNF)‐α and interleukin (IL)−6, while M2 type expresses pro‐healing cytokines such as IL‐10, vascular endothelial growth factor (VEGF), and transforming growth factor (TGF)‐β.^[^ ^8^, ^9^ ^]^ Since infected wound microenvironments face high levels of inflammation and oxidative stress, which can inhibit the polarization of macrophages from the M1 to M2 type, the wounds hardly undergo the proliferation stage and easily form chronic wounds. An increasing number of studies have shown that the phenotypic regulation of macrophages from the M1 to M2 type can effectively promote wound healing by decreasing the level of inflammation and promoting the regeneration of granulation tissues and vascularization.^[^ ^10^, ^11^, ^12^ ^]^ Hence, researchers should raise concerns regarding immune regulation, except for anti‐bacteria. Given the specificity and complexity of the wound microenvironments of MDR bacteria‐infected wounds, a strategy that exhibits antibacterial, immune regulation, anti‐inflammation, and antioxidation properties should be more effective than traditional methods for MDR bacteria‐infected wound healing.

Currently, antibacterial materials are mainly developed into nanomaterials and antimicrobial peptides (AMPs).^[^ ^13^, ^14^ ^]^ The potential toxicity of metal ions toward tissues and hyperthermia damage (>50 °C) to normal tissues from nanomaterials have raised concerns in public.^[^ ^15^, ^16^, ^17^ ^]^ In contrast, AMPs as natural immune defense substances have broad antibacterial activity, negligible toxicity to eukaryotes, and hardly induce the formation of drug‐resistant bacteria.^[^ ^18^, ^19^ ^]^ For instance, KR‐12 peptide is the smallest antibacterial motif of human cathelicidin LL‐37, and has robust antibacterial activity. It also modulates the inflammatory response and accelerates the re‐epithelialization process.^[^ ^20^, ^21^ ^]^ However, the biggest disadvantage of AMPs is that it easily decompose in physiological conditions.^[^ ^18^ ^]^ Cryogel is a type of porous material that usually exhibit great flexibility, water absorption capacity, and gas permeability, is an excellent drug delivery platform, and has been widely applied to the field of wound healing.^[^ ^22^, ^23^, ^24^ ^]^ The main body of the cryogel is build using hyaluronic acid (HA), a natural linear polysaccharide distributed in ECM of hydrated tissues, which possesses good biocompatibility, biodegradability, and strong moisture retention ability, and also contains abundant active groups.^[^ ^25^ ^]^ In addition, HA is a responsive degradation material that can be decomposed by hyaluronidase produced by bacteria.^[^ ^26^ ^]^ Our previous study showed that the natural polyphenol tannic acid (TA) is an ideal candidate for anti‐oxidation, and can also exhibit antibacterial and anti‐inflammatory effects to an extent.^[^ ^23^, ^27^, ^28^ ^]^

**Scheme 1:**
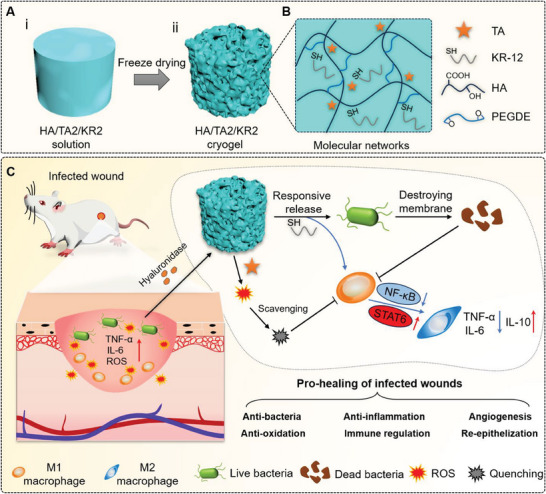
HA/TA2/KR2 cryogel cures the infected wound. A) Illustration of the HA/TA2/KR2 cryogel fabrication. The solution state (i) and the scaffold state (ii). B) Molecular networks of the HA/TA2/KR2 cryogel in (A). C) The combined effect of the HA/TA2/KR2 cryogel for promoting healing of MDR bacteria‐infected wounds. Some elements are cited from our previous work and the copyright permission has been achieved.^[^ ^28^ ^]^

Herein, we fabricated an HA/TA2/KR2 cryogel composed of HA, poly (ethylene glycol) diglycidyl ether (PEGDE), TA, and KR‐12 via chemical crosslinking and freeze‐drying (**Scheme** **1**A). HA and PEGDE formed the main molecular networks via ether bonds, which interacted with the TA molecules through intermolecular hydrogen bonds (Scheme 1B). The natural polyphenol TA serves two purposes: 1) improving the mechanical strength of the cryogel and 2) cooperating with KR‐12 to control infection, inflammation, and oxidative stress. In MDR bacteria‐infected wounds, the HA/TA2/KR2 cryogel could responsively release KR‐12 to clear bacteria and modulate macrophages to the M2 type by inhibiting Nuclear factor kappa B (NF‐κB) and activating the Janus kinase‐Signal transducer activator of transcription (JAK‐STAT6) signaling pathway. In addition, TA and KR‐12 could synergistically scavenge overexpressed reactive oxygen species (ROS) and inflammatory cytokines in wound tissues, minimizing damage to the normal ECM and cells. Therefore, KR‐12 and TA could reshape the infected wound microenvironment, achieving various functions, including antibacterial, anti‐inflammatory, and anti‐oxidation, resulting in fast re‐epithelialization and angiogenesis. Finally, the HA/TA2/KR2 cryogel exhibited excellent pro‐healing performance against MDR bacteria‐infected wounds, attributing a synergistic effect (Scheme 1C).

## 2 Results

### 2.1 Fabrication and Characterization of HA/TA2/KR2 Cryogel

**Figure 1:**
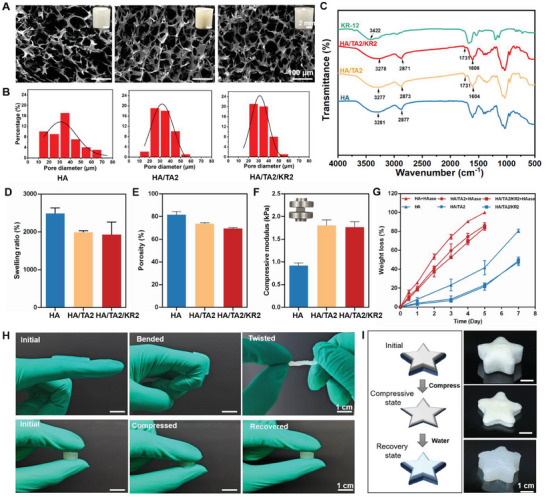
Morphological, physical, and mechanical properties of cryogels. A) SEM images of the HA, HA/TA2, and HA/TA2/KR2 cryogels. Insets: Images of real cryogels. B) Pore diameter distributions of HA, HA/TA2, and HA/TA2/KR2 cryogels, corresponding to (A). C) FTIR spectra of the HA, HA/TA2, and HA/TA2/KR2 cryogels. D,E) Swelling ratios and porosities of the HA, HA/TA2, and HA/TA2/KR2 cryogels. F) Compressive moduli of the HA, HA/TA2, and HA/TA2/KR2 cryogels. G) Degradability of the HA, HA/TA2, and HA/TA2/KR2 cryogels in different solutions, with and without hyaluronidase (HAase). H) Mechanical properties of the HA/TA2/KR2 cryogel, including flexibility and elasticity. I) Shape memory properties of the HA/TA2/KR2 cryogels based on the porous structures and water absorbance.

Cryogels composed of HA, PEGDE, TA, and KR‐12 were fabricated using a two‐step method comprising a chemical crosslinking step and a freeze‐drying step (molecular structures in Figure S1, Supporting Information). TA, a natural polyphenol as an antioxidant, may be cytotoxic at high concentrations. First, we used HA/TA cryogels containing TA concentrations of 0.1%, 0.2%, and 0.5% (w/v) to co‐incubate with NIH3T3 cells for 3 days. The result showed that 0.1% and 0.2% (w/v) TA did not influence the viability of NIH3T3 cells, but 0.5% TA (w/v) was cytotoxic (Figure S2, Supporting Information). Since TA is a concentration‐related antioxidant, we chose 0.2% (w/v) TA (denoted as HA/TA2) as the antioxidant ingredient for the following biological experiments and binding with the KR‐12 peptide. Next, the morphology and mechanical and physical properties of HA, HA/TA2, and HA/TA2/KR2 cryogels were characterized. Scanning electron microscopy (SEM) images showed that HA, HA/TA2, and HA/TA2/KR2 cryogels exhibited 10–80 µm porous structures in the pore diameter, of whom HA/TA2/KR2 cryogel had the best homogeneity in the pore size (**Figure** **1**A,B). The trends in pore structure and diameter depended on the addition of TA and KR‐12, indicating that intermolecular interactions between TA or KR‐12 and HA dominated the differences among the cryogels (Figure S3A,B, Supporting Information). Next, Fourier transform infrared (FTIR) spectroscopy was used to acquire the characteristic absorption peaks and explore the intermolecular interactions in the cryogels. All cryogels exhibited characteristic absorption peaks at 3700–3000 cm^−1^, the maximum absorption peaks in the HA/TA2 and HA/TA2/KR2 cryogels underwent a redshift relative to the HA cryogel (Figure 1C), which may have been caused by intermolecular hydrogen bonds. Moreover, the stretch and variation peak of carbonyl (C═O) at 1731 cm^−1^ appeared in HA/TA2 and HA/TA2/KR2 cryogels, revealing the presence of ester bonds in these cryogels, originating from TA (Figure S1C, Supporting Information). Additionally, the characteristic absorption peaks of the HA/TA2/KR2 cryogel were similar to those of the HA/TA2 cryogel, whereas the characteristic absorption peaks of KR‐12 were completely shielded by HA, indicating no chemical bond formation (Figure 1C). In summary, the FTIR spectra demonstrated that the addition of TA can form intermolecular hydrogen bonds with HA, whereas KR‐12 is simply dispersed in HA molecular networks.

Subsequently, the mechanical and physical properties of the cryogels, including the imbibition, porosity, and compressive modulus, were characterized. Swelling experiments showed that all cryogels possessed a high water‐absorbing ability; among them, the HA/TA2/KR2 cryogel exhibited a swelling ratio of 1929.8% (Figure 1D). Porosity measurements showed that the HA/TA2/KR2 cryogel had a high porosity of ≈70%, which was attributed to its porous structure (Figure 1E). As shown in Figure 1F, the mean compressive moduli of the HA, HA/TA2, and HA/TA2/KR2 cryogels were 0.9, 1.8, and 1.7 kPa, respectively. Compressive tests further proved that the addition of TA enhanced the mechanical properties of the cryogels. Additional experiments showed that the morphology and physical and mechanical properties of the cryogels were positively related to the TA content (Figure S3, Supporting Information). Combined with the FTIR spectra and molecular structures of TA and HA, the TA molecules acted as new crosslinkers to bond with HA networks through intermolecular hydrogen bonds,^[^ ^25^ ^]^ which is why the addition of TA enhanced the crosslinking density of the HA cryogel and improved its mechanical performance. Since the HA/TA2/KR2 cryogel exhibited a porous structure at the 1.7 kPa modulus level, it proposed great flexibility, elasticity, and shape‐memory capability. Figure 1H shows that the wet HA/TA2/KR2 cryogel could be bended by 90° and was severely twisted and compressible. Another interesting property was the shape‐memory capability, where the severely deformed HA/TA2/KR2 cryogel immediately recovered its initial shape after absorbing sufficient water (Figure 1I). Due to its excellent mechanical properties and water absorption capacity, the HA/TA2/KR2 cryogel was anticipated as a hemostatic material. Two classical hemostasis models (rat liver and tail) were selected to characterize the hemostatic properties of the HA/TA2/KR2 cryogel. The liver blood loss was 293.7 mg in the HA/TA2/KR2 group, which was remarkably less than 2126.7 mg in the control group and 901.0 mg in the gauze group, and also slightly less than 409.3 mg in the alginate Ag group (Figure S4A–C, Supporting Information). The hemostasis time of the liver in the HA/TA2/KR2 group was less than 60 s, which was better than that of gauze and alginate Ag. Rat‐tail hemostasis tests were consistent with the trend in the liver hemostasis tests, where the HA/TA2/KR2 group showed the least blood loss of ≈160 mg and the shortest hemostasis time of ≈48 s, achieving better hemostasis performance than alginate Ag (Figure S4D–F, Supporting Information). The experimental results of blood loss and hemostasis time showed that the HA/TA2/KR2 cryogel exhibited better hemostasis performance than the commercial wound dressing alginate Ag. In conclusion, the mechanical and physical properties proved that the HA/TA2/KR2 cryogel is suitable as an infected wound dressing.

Finally, we tested the degradation behavior of the cryogels by soaking them in phosphate buffered saline (PBS) with or without hyaluronidase (HAase: 100 U mL^−1^) for seven days.^[^ ^29^, ^30^ ^]^ Figure 1G reveals that the HA cryogel in pure PBS was degraded by up to 80.6% on the 7th day, whereas simultaneously, the HA/TA2 and HA/TA2/KR2 cryogels only lost 47.5% and 49.0% of their weight, respectively, which meant that crosslinking with TA could significantly decrease the degradation rate of the cryogels. Upon treatment with hyaluronidase, all cryogels degraded significantly faster, and the weight loss ratios of HA, HA/TA2, and HA/TA2/KR2 were 100.0%, 86.3%, and 84.0%, respectively, on the 5th day. Because HA can be broken down by HAase, the release behavior of the active ingredients, including TA and KR‐12, was further tested. Indeed, the degradation rate of HA depended on the concentration of HAase; thus, the concentrations of HAase at 0 (PBS solution), 50, 100, and 200 U mL^−1^ were chosen to conduct the release experiments for 96 h. With an increase in HAase, TA was released quickly and reached a maximum release ratio of ≈60.9% (Figure S5, Supporting Information). KR‐12 showed a similar release behavior, in which the release ratio increased with the concentration of HAase, but reached a plateau at 48 h and the maximum release ratio was ≈66.8% (Figure S6, Supporting Information). Furthermore, the release behavior of KR‐12 related to the concentration of TA was checked simultaneously. The results showed that both the maximum release ratio and the release rate decreased with an increase in TA in HA/TA2/KR2 cryogels, indicating that a higher crosslinking degree would inhibit the release of KR‐12 (Figure S7, Supporting Information).

### 2.2 In Vitro Cytocompatibility, Blood Compatibility, and Migration Promoting Capacity of HA/TA2/KR2 Cryogel

**Figure 2:**
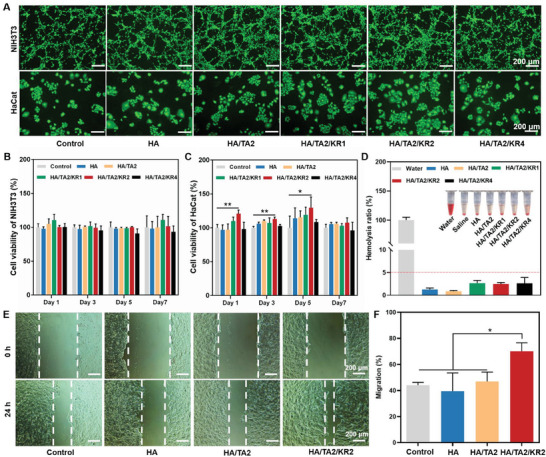
Cytocompatibility, blood compatibility, and pro‐migration ability of cryogels. A) Live/dead cell staining images of NIH3T3 and HaCat cells after co‐incubation with pure medium and HA, HA/TA2, HA/TA2/KR1, HA/TA2/KR2, and HA/TA2/KR4 cryogles on the 3rd day. B,C) Viability of B) NIH3T3 and C) HaCat cells after co‐incubation with pure medium and cryogels for 1, 3, 5, and 7 days (*n* = 4). D) Hemolysis ratios of various cryogels (*n* = 3); inset: images of erythrocyte suspensions. E) The migration capability of HaCat cells co‐incubated with pure medium, HA, HA/TA2, and HA/TA2/KR2 cryogels after 24 h. F) Migration ratio of HaCat cells in the control, HA, HA/TA2, and HA/TA2/KR2 groups (*n* = 3). *p* < 0.05 (“^*^”) and *p* < 0.01 (“^**^”).

Excellent biocompatibility is a prerequisite of wound dressings. Because antimicrobial peptides at high concentrations may be toxic, we mainly tested the potential cytotoxicity of cryogels in relation to the concentration of the KR‐12 peptide. The HA/TA2/KR1, HA/TA2/KR2, and HA/TA2/KR4 cryogels with 1.0, 2.0, and 4.0 mg mL^−1^ KR‐12, respectively, and the HA and HA/TA2 cryogels were tested via a live/dead cell staining method and a Cell Counting Kit 8 (CCK8) test for NIH3T3 and HaCat cells. Fluorescence images of NIH3T3 and HaCat cells demonstrated that the HA, HA/TA2, HA/TA2/KR1, HA/TA2/KR2, and HA/TA2/KR4 cryogels were almost non‐toxic, as shown in **Figure** **2**A. The CCK8 experiments furthermore quantified the cytocompatibility of the cryogels for seven days. The results revealed that the impairment of NIH3T3 cells in the HA, HA/TA2, HA/TA2/KR1, HA/TA2/KR2, and HA/TA2/KR4 cryogels was negligible during the seven days (Figure 2B). Consistent with NIH3T3 cells, the growth of HaCat cells over seven days was not impaired by the cryogels (Figure 2C). In particular, the HA/TA2/KR2 cryogel significantly promoted the proliferation of HaCat cells compared with the control group for the first 5 days. In addition, the cytocompatibility of free TA and KR‐12 was conducted for seven days. It showed that once the mass of free KR‐12 increased to 200 µg, a significant depression in cell viability was observed in both NIH3T3 and HaCat cells (Figure S8, Supporting Information). This cytotoxic effect was consistent with that observed in a previous study.^[^ ^21^ ^]^ Hemolysis is another important indicator of biocompatibility. The hemolysis experiment revealed that the hemolysis ratios of HA, HA/TA2, HA/TA2/KR1, HA/TA2/KR2, and HA/TA2/KR4 cryogels were between 0.9% and 2.7%, which is far below the safe line of 5% (Figure 2D). In addition, a migration experiment of HaCat cells demonstrated that the HA/TA2/KR2 cryogel effectively promoted the migration of HaCat cells, which was not limited to good biocompatibility. As shown in Figure 2E, HaCat cells incubated with HA/TA2/KR2 cryogel showed the highest migration rate. Quantitative results demonstrated that the HA/TA2/KR2 group reached a migration ratio of 70.1% after 24 h, whereas the control, HA, and HA/TA2 groups only achieved 39.5–47.0% migration ratio (Figure 2F). All results revealed that the HA/TA2/KR2 cryogel possessed excellent biocompatibility and pro‐migration capability, meeting the standards for wound dressings.

### 2.3 In Vitro Antibacterial Property and Mechanism of HA/TA2/KR2 Cryogel

**Figure 3:**
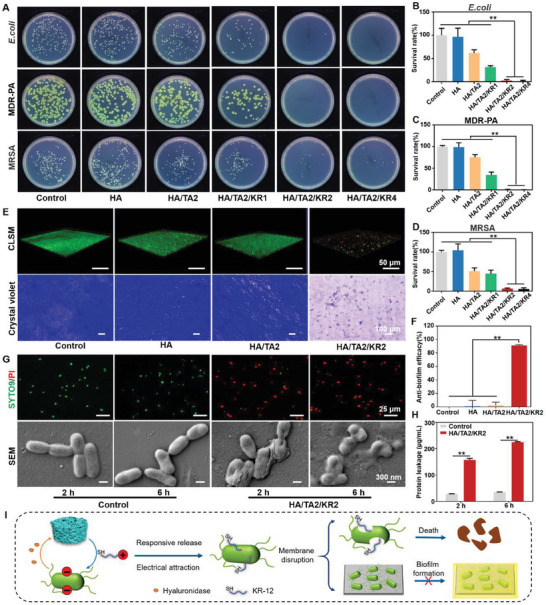
In vitro antibacterial property and mechanism. A) Images of bacterial colonies of *E. coli*, MDR‐PA, and MRSA after pretreatment with pure medium, HA, HA/TA2, HA/TA2/KR1, HA/TA2/KR2, and HA/TA2/KR4 cryogels. B–D) Survival rates of *E. coli*, MDR‐PA, and MRSA in different groups (*n* = 3), corresponding to (A). E) CLSM and crystal violet images of MDR‐PA biofilms in the control, HA, HA/TA2, and HA/TA2/KR2 groups. F) Anti‐biofilm efficacy of HA, HA/TA2, and HA/TA2/KR2 groups (*n* = 3), corresponding to (E). G) Live/dead stained bacteria and corresponding SEM images in the control and HA/TA2/KR2 groups at 2 and 6 h. H) Protein leakage contents of bacteria in the control and HA/TA2/KR2 groups at 2 and 6 h (*n* = 3), corresponding to (G). I) Antibacterial mechanism of HA/TA2/KR2 cryogels. *p* < 0.05 (“^*^”) and *p* < 0.01 (“^**^”).

Antibacterial properties are another important index of infected wound dressings. KR‐12 in the cryogel mainly played an antibacterial role. Since HAase can be produced by bacteria,^[26^ ^]^ the enzyme‐responsive release behavior of KR‐12 was first tested by co‐incubation with or without MDR‐PA. Results unveiled that the presentation of bacteria could evidently promote the release process of KR‐12, and the equilibrium concentration of KR‐12 (323.5 µg mL^−1^) was twice that of without bacteria (Figure S9, Supporting Information). To mimic the bacteria‐infected microenvironment, different concentrations (0 [PBS solution], 0.2 × 10^8^, 1 × 10^8^, and 5×10^8^ CFU/mL) of bacteria were inoculated to the wounds to study the release of KR‐12 from HA/TA2/KR2. The release ratio of KR‐12 increased from 28.7% to 42.3%, indicating concentration‐dependent release behavior (Figure S10, Supporting Information). This responsive release characteristic was consistent with the results of Figure S6 (Supporting Information) and was beneficial for improving the bactericidal efficiency of KR‐12 and reducing its side effects. Subsequently, the gram‐negative bacterium *Escherichia coli* (*E. coli)* and the gram‐positive bacteria MDR‐PA and MRSA were chosen as classical objects to evaluate the practical antibacterial efficacy of the cryogels. After incubation with HA, HA/TA2, HA/TA2/KR1, HA/TA2/KR2, and HA/TA2/KR4 cryogels, the bacterial colonies on the agar culture plates showed significant differences among the groups (**Figure** **3**A). KR1, KR2, and KR4 denoted that the cryogels were fabricated with 1.0, 2.0, and 4.0 mg mL^−1^ of KR‐12, respectively. Without KR‐12 and TA, the HA cryogel could not kill any bacteria, based on images of bacterial colonies and quantitative analysis. The addition of TA endowed the HA/TA2 cryogel with a certain degree of antibacterial property for three kinds of bacteria, achieving a kill ratio of 24.5–48.8%. When KR‐12 was combined with TA and HA, antibacterial effect followed the order of HA/TA2/KR4 ≈ HA/TA2/KR2 > HA/TA2/KR1, depicting that 2.0 mg mL^−1^ was the best KR‐12 concentration. The antibacterial efficacy was concentration‐dependent; HA/TA2/KR1 achieved a killing ratio of only 55.3–68.6%, whereas HA/TA2/KR2 and HA/TA2/KR4 both showed a killing ratio of >93% (Figure 3B–D). Moreover, KR‐12 exhibited a better antibacterial ratio against gram‐negative bacteria than against gram‐positive bacteria. For instance, HA/TA2/KR2 almost completely killed *E. coli* and MDR‐PA with scavenging ratios of 97.7% and 99.6%, respectively, but killed MRSA with a killing ratio of 93.4% (Figure 3B–D). Because HA/TA2/KR2 showed the best cytocompatibility and equal antibacterial activity to HA/TA2/KR4, it was selected as the optimal cryogel for the following experiments.

Given the degradation of KR‐12, the long‐term antibacterial properties of HA/TA2/KR2 and KR‐12 were evaluated. The results revealed that both HA/TA2/KR2 and KR‐12 maintained the antibacterial ratio of MDR‐PA at ≈100% for 7 days, whereas only HA/TA2/KR2 maintained the antibacterial ratio of MRSA at 96.7–99.3% (Figure S11, Supporting Information). Single KR‐12 rapidly decreased the antibacterial capability on day 5 owing to the degradation of KR‐12 and bacterial proliferation.

Bacterial biofilms are a survival barrier that can resist drugs and antibiotics to heavily suppress wound healing.^[^ ^31^ ^]^ Therefore, preventing the formation of bacterial biofilms is closely related to the process of promoting infected wound healing. As shown in Figure 3E, confocal laser scanning microscopy (CLSM) images of live/dead bacterial cell staining and optical images of crystal violet staining clearly revealed the formation of MDR‐PA biofilms in control, HA, and HA/TA2 groups, whereas no obvious biofilms were formed in the HA/TA2/KR2 group. Quantitative results demonstrated that the anti‐biofilm efficacy of the HA and HA/TA2 groups was below 5%, whereas that of the HA/TA2/KR2 group reached 91.3%, indicating excellent anti‐biofilm properties (Figure 3F). All experimental results showed that KR‐12 has a wide antimicrobial spectrum and an excellent anti‐biofilm effect. Based on the molecular structure (Figure S1D, Supporting Information), KR‐12 contained amino groups presenting positive electricity, which may break the bacterial plasma membrane and induce bacterial death. Next, we used fluorescent probes and SEM to explore the cause of bacterial death after co‐incubation with the HA/TA2/KR2 cryogel or no treatment. In contrast to that in the control group, widespread bacterial death began at 2 h in the HA/TA2/KR2 group (Figure 3G). The corresponding SEM images demonstrated that the bacterial cell membranes started breaking at 2 h, and a large amount of content was released into the substrates at 6 h. Subsequently, the amount of protein leakage was measured by BCA protein assays. In the HA/TA2/KR2 group, the protein leakage concentrations were 157.3 and 224.7 µg mL^−1^ after 2 and 6 h, respectively. Simultaneously, the protein leakage in the control group was always maintained at 28.6–35.4 µg mL^−1^ (Figure 3H). Therefore, the HA/TA2/KR2 cryogel exhibited excellent anti‐MDR bacterial capability via a physical damage mechanism by disrupting the bacterial cell membranes (Figure 3I).

### 2.4 In Vitro Antioxidation and ROS Scavenging Capacity of HA/TA2/KR2 Cryogel

**Figure 4:**
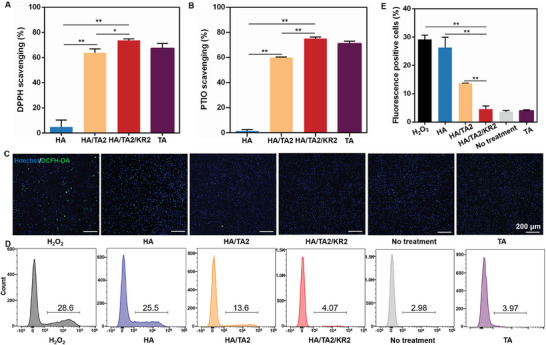
Free radical scavenging and ROS scavenging property. A) DPPH∙ scavenging activity in the HA, HA/TA2, HA/TA2/KR2, and TA groups (*n* = 3). B) PTIO∙ scavenging activity in the HA, HA/TA2, HA/TA2/KR2, and TA groups (*n* = 3). C) Fluorescence images of ROS distribution of BMDMs in the H_2_O_2_ (positive control), HA, HA/TA2, HA/TA2/KR2, no treatment (negative control), and TA groups. D) ROS flow images of the H_2_O_2_, HA, HA/TA2, HA/TA2/KR2, no treatment, and TA groups. E) ROS^+^ cell ratios in the various groups (*n* = 3) corresponding to (D). *p* < 0.05 (“^*^”) and *p* < 0.01 (“^**^”).

Bacterial colonization in wound tissues not only induces an inflammatory response but also causes an overexpression of ROS. It is well known that overexpressed ROS in infected wound tissues generally damages normal cells and further induces heavy inflammation, forming a vicious circle.^[^ ^32^ ^]^ Therefore, antioxidation is essential for infected wound healing. Because the HA/TA2/KR2 cryogel contained the natural antioxidant TA, it could effectively clear various free radicals. The HA, HA/TA2, HA/TA2/KR2, and free TA (mass: 200 µg, equivalent to the TA in cryogel) groups were tested using DPPH∙ and PTIO∙ assay kits. The results showed that the scavenging ratios of DPPH∙ and PTIO∙ in the HA/TA2 group reached 63.8% and 60.0%, respectively. The HA/TA2/KR2 group showed a good scavenging capability as the free TA group, reaching scavenging ratios of ≈70% for DPPH∙ and PTIO∙ (**Figure** **4**A,B). Free radical scavenging experiments showed that the scavenging capability of TA in the HA/TA2 cryogel was weakened to some extent; however, the addition of KR‐12 compensated for this loss. The increase in the free radical scavenging ratio in the HA/TA2/KR2 group can be attributed to the cysteine residues in KR‐12 (Figure S1D, Supporting Information).^[^ ^33^ ^]^ In addition, the antioxidant function was tested through in vitro cell experiments. H_2_O_2_ (0.5 mm) was used to stimulate bone marrow‐derived macrophages (BMDMs), and ROS expression in cells was checked via a fluorescent probe (DCFH‐DA). ROS fluorescence images revealed that ROS in the HA/TA2 group was almost completely quenched, and that in the HA/TA2/KR2 and TA groups was completely quenched to the level of the negative group (no treatment) (Figure 4C). Furthermore, flow cytometry was used to quantitatively measure the ROS expression in the positive control, HA, HA/TA2, HA/TA2/KR2, TA, and negative control groups. As shown in Figure 4D,E, ROS ratios of the positive control, HA, HA/TA2, HA/TA2/KR2, TA, and negative control groups were 29.1%, 26.3%, 13.7%, 4.6%, 3.6%, and 4.0%, respectively. Both antioxidation and ROS scavenging experiments proved that the combination of TA and KR‐12 reached the best performance. To confirm the possible synergistic effect of TA and KR‐12,^[^ ^34^, ^35^ ^]^ the ROS scavenging capability of four groups (control, HA/TA2, HA/KR2, and HA/TA2/KR2) was tested using the fluorescent probe. HA/TA2 refers to the single role of TA, HA/KR2 refers to the single role of KR‐12, and HA/TA2/KR2 refers to the synergistic role of TA and KR‐12. As shown in Figure S12 (Supporting Information), HA/TA2/KR2 could quench ≈91.4% of ROS relative to the control, which was significantly higher than HA/TA2 (quench ratio of 61.1%) and HA/KR2 (quench ratio of 29.4%), and more than the sum of HA/TA2 and HA/KR2 (90.5%). Hence, the HA/TA2/KR2 cryogel exhibited excellent antioxidation and ROS‐scavenging capabilities, which were attributed to the synergistic effect of TA and KR‐12.

### 2.5 In Vitro Anti‐Inflammation and Macrophage Phenotype Regulation of HA/TA2/KR2 Cryogel

**Figure 5:**
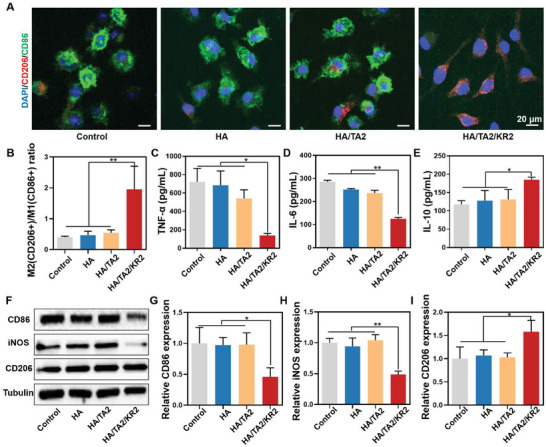
Regulation of macrophage phenotypes and anti‐inflammation effect. A) Immunofluorescence images of BMDMs from the control, HA, HA/TA2, and HA/TA2/KR2 groups. B) Ratios of CD206^+^/CD86^+^ BMDMs in the control, HA, HA/TA2, and HA/TA2/KR2 groups (*n* = 3). C–E) Expression levels of TNF‐α, IL‐6, and IL‐10 in the control, HA, HA/TA2, and HA/TA2/KR2 groups (*n* = 3). F) Western blotting images of CD86, iNOS, CD206, and Tubulin in the control, HA, HA/TA2, and HA/TA2/KR2 groups. (G‐I) Relative expression of CD86, iNOS, and CD206 in the control, HA, HA/TA2, and HA/TA2/KR2 groups (n = 3), corresponding to (F). P < 0.05 (“*”) and P < 0.01 (“**”).

Macrophages are the main immune cells in skin wound tissues, and can secrete pro‐inflammatory (TNF‐α and IL‐6) and anti‐inflammatory (IL‐10) cytokines depending on phenotypes. M1 type macrophages in bacteria‐infected wounds can clear and swallow bacteria; however, overactivated M1 macrophages strongly aggravate the inflammatory response and delay collagen deposition to hinder wound healing.^[^ ^36^ ^]^ Therefore, the regulation of macrophage phenotype is also important for promoting bacteria‐infected wound healing. First, we tested the regulatory capability of the cryogels by incubating lipopolysaccharide (LPS)‐activated M1 type BMDMs with the cryogels. Immunofluorescence images of BMDMs showed that the M1 type maker CD86 was significantly decreased in the HA/TA2/KR2 group, in contrast to the control, HA, and HA/TA2 groups. In contrast, the M2 type maker CD206 in the HA/TA2/KR2 group grew significantly (**Figure** **5**A). Statistical analysis of the CD206^+^/CD86^+^ ratio revealed that the HA and HA/TA2 groups were approximately at the level of 0.5, similar to the control group, whereas the HA/TA2/KR2 group increased to 1.9 (Figure 5B). These experimental results demonstrate that the fabricated HA/TA2/KR2 cryogel may regulate the phenotype of macrophages from M1 to M2 in an LPS‐stimulated microenvironment. To further verify the regulation effect of the HA/TA2/KR2 cryogel, classical pro‐inflammatory (TNF‐α and IL‐6) and anti‐inflammatory (IL‐10) cytokines were measured via enzyme‐linked immunosorbent assay (ELISA) kits. As shown in Figure 5C,D, pro‐inflammatory cytokines TNF‐α and IL‐6 decreased to 138.8 and 123.9 pg mL^−1^, respectively, in the HA/TA2/KR2 group, compared to 722.7 and 285.6 pg mL^−1^, respectively, in the control group. In addition, the anti‐inflammatory cytokine IL‐10 in the HA/TA2/KR2 group increased to 184.9 pg mL^−1^ from 117.7 pg mL^−1^ in the control group (Figure 5E). However, HA and HA/TA2 cryogels did not markedly change the expression level of TNF‐α, IL‐6, and IL‐10. Because of the presence of synergistic antioxidation between TA and KR‐12, the synergistic anti‐inflammation effect between TA and KR‐12 was also explored (Figure S13, Supporting Information). The ELISA results showed that HA/TA2/KR2 could reduce 74.6% and 61.9% of TNF‐α and IL‐6 relative to the control, respectively, both of which were more than the sum of HA/TA2 and HA/KR2 (TNF‐α:51.9%; IL‐6: 49.5%). This data confirmed the synergistic effect of TA and KR‐12 on the inhibition of pro‐inflammatory cytokines. Subsequently, western blotting (WB) was performed to further confirm these phenomena based on the relative expression of CD86, CD206, and inducible nitric oxide synthase (iNOS). WB results revealed that the HA/TA2/KR2 cryogel significantly downregulated CD86 and iNOS levels and upregulated CD206 level (Figure 5F). Quantitative analysis showed that the relative expression levels of CD86 and iNOS decreased by 2 times, while that of CD206 increased by 1.5 times in the HA/TA2/KR2 group (Figure 5G–I). The excellent regulatory effect of HA/TA2/KR2 also originated from the synergistic effect of TA and KR‐12 (Figure S14, Supporting Information). Based on the experimental results, we found that the HA/TA2/KR2 cryogel could regulate M1 type macrophages to M2 type macrophages and suppress the inflammatory reaction, indicating that the cryogel possessed a phenotypic regulation effect.

### 2.6 In Vivo Promotion of Infected Wound Healing via HA/TA2/KR2 Cryogel

**Figure 6:**
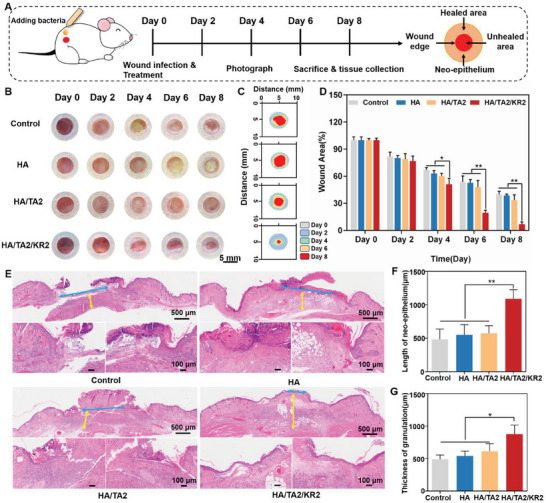
MDR‐PA bacteria‐infected wound healing. A) Schematic of the experimental procedures for MDR‐PA bacteria‐infected wound healing and definition of the healed wound area. B) Images of MDR‐PA bacteria‐infected wounds in the control, HA, HA/TA2, and HA/TA2/KR2 groups during 8 days. C) Overlay images of the wound areas in the control, HA, HA/TA2, and HA/TA2/KR2 groups over time. D) Wound healing trends in the control, HA, HA/TA2, and HA/TA2/KR2 groups on days 0, 2, 4, 6, and 8 (*n* = 5). E) H&E‐stained images of wound tissues in the control, HA, HA/TA2, and HA/TA2/KR2 groups on day 8. The blue and yellow arrows denote the gap between the neo‐epithelium at both ends and the thickness of the granulation tissue, respectively. F) The length of neo‐epithelium in the control, HA, HA/TA2, and HA/TA2/KR2 groups (*n* = 4). G) Thickness of granulation tissue in the control, HA, HA/TA2, and HA/TA2/KR2 groups (*n* = 4). *p* < 0.05 (“^*^”) and *p* < 0.01 (“**”).

Owing to the excellent performance of the HA/TA2/KR2 cryogel in biocompatibility, antibacterial, antioxidation, and anti‐inflammation tests, it meets the standards of an ideal wound dressing and may effectively promote MDR bacteria‐infected wound healing. BALB/c mice were used to build MDR‐PA bacteria‐infected wounds on day 0, then the pictures were captured on days 2, 4, 6, and 8. Mice were sacrificed, and wound tissues were collected on days 4 and 8 for histological analysis (**Figure** **6**A). We divided the MDR‐PA bacteria‐infected wounds into four groups: control (gauze), HA, HA/TA2, and HA/TA2/KR2. As shown in Figure 6B, a large amount of purulent exudation was observed in the wounds of the control, HA, and HA/TA2 groups on days 4, 6, and 8, whereas only mild redness was observed in the wounds of the HA/TA2/KR2 group. Notably, a newly formed epidermis was observed on the surface of the wounds treated with the HA/TA2/KR2 cryogel. As shown in Figure 6C, the overlay images of the wound areas clearly revealed trends and differences in wound healing among the groups. Statistical results of the wound areas proved that the HA/TA2/KR2 cryogel could significantly promote infected wound healing compared with the other groups (Figure 6D). In addition, the HA/TA2 cryogel only had a limited repair effect on the infected wounds compared to the control and HA cryogels. In vivo experiments demonstrated that the HA/TA2/KR2 cryogel met the design and usage requirements. Furthermore, histological techniques were used to analyze the differences and reasons for infected wound healing among the groups based on hematoxylin and eosin (H&E) staining. When comparing the H&E staining of wound tissues among the groups on the 8th day, the regeneration degrees of epithelization and granulation tissues were the main evaluation indicators. As shown in Figure 6E, the blue and yellow arrows denote the gap between the neo‐epithelium at both ends and the thickness of the granulation tissues in the wounds, respectively. On the 8th day, the degree of epithelization regeneration in the control group was the lowest, which was slightly better in the HA and HA/TA2 groups, and the best in the HA/TA2/KR2 group (Figure 6E,F). Similarly, the thickness of the granulation tissues followed the order HA/TA2/KR2 > HA/TA2 & HA > control (Figures 6E,G). The in vivo tests proved that the HA/TA2/KR2 cryogel is an excellent dressing for infected wounds.

### 2.7 In Vivo antibacterial, Antioxidative, Anti‐Inflammatory, and Macrophage Phenotype Regulation of HA/TA2/KR2 Cryogel

**Figure 7:**
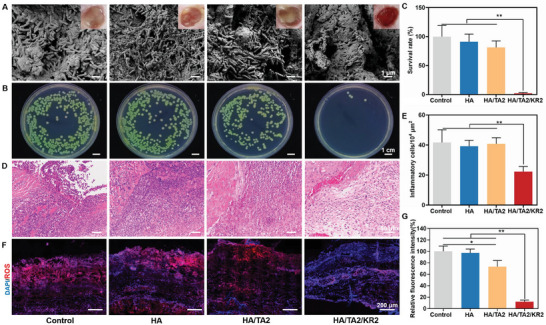
In vivo antibacterial, anti‐inflammatory, and ROS scavenging activity. A) SEM images of MDR‐PA bacteria in wound tissues from the control, HA, HA/TA2, and HA/TA2/KR2 groups on day 4. B) MDR‐PA bacterial colonies from various groups corresponding to (A). C) Survival rates of MDR‐PA in various groups (*n* = 3). D) H&E staining of wound tissues from various groups on day 4. E) Inflammatory cell density (*n* = 3) corresponding to (D). F) Immunofluorescence images of ROS in the wound tissues. G) Relative fluorescence intensities of ROS (*n* = 3). *p* < 0.05 (“^*^”) and *p* < 0.01 (“^**^”).

To explain why the HA/TA2/KR2 cryogel exhibited excellent performance for MDR bacteria‐infected wound healing, wound tissues were collected on the 4th day to perform histological sections and staining, SEM, and bacterial plate culture, to check for antibacterial, anti‐inflammatory, and antioxidative effects in vivo. The SEM images in **Figure** **7**A clearly reveal the distribution of MDR‐PA bacteria in the wound tissues from different groups, with the number of bacteria in the HA/TA2/KR2 group being the lowest. After bacterial coating of the plates and culture, bacterial colonies in the HA/TA2/KR2 group were less than in the other groups (Figure 7B). The colony count results revealed that the survival rates of MDR‐PA bacteria were 2.2%, 81.3%, and 91.3% in the HA/TA2/KR2, HA/TA2, and HA groups, respectively (Figure 7C). H&E staining of the wound tissues was performed to analyze inflammatory cell infiltration at the wound sites. Figure 7D,E shows that fewer inflammatory cells were present in the HA/TA2/KR2 group, ≈20 × 104 cells/µm^2^, compared to 40 × 10^4^ cells/µm^2^ in the other groups. We also measured ROS levels in wound tissues using frozen tissue sections and immunofluorescence staining of ROS. As shown in Figure 7F, the ROS area in the HA/TA2/KR2 group sharply decreased compared with that in the other groups. By measuring the relative fluorescence intensity of ROS, we found that the clear ratio of ROS was nearly 90% in the HA/TA2/KR2 group, in contrast to that in the control group (Figure 7G). In vivo animal experiments showed that the HA/TA2/KR2 cryogel had excellent antibacterial, antioxidative, and anti‐inflammatory properties.

**Figure 8:**
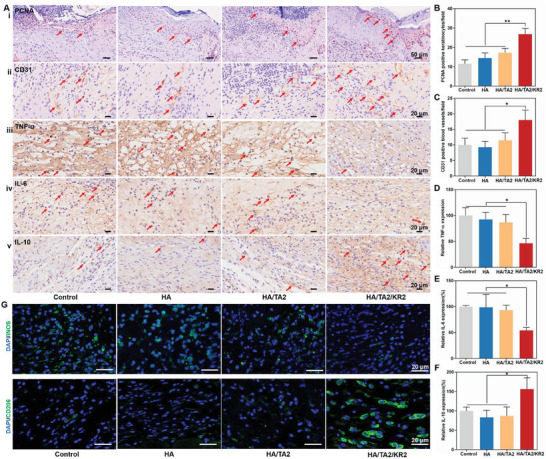
Immunohistochemical and immunofluorescence staining of wound tissues. A) Immunohistochemical staining of PCNA (Ai), CD31 (Aii), TNF‐α (Aiii), IL‐6 (Aiv), and IL‐10 (Av) in the wound tissues from the control, HA, HA/TA2, and HA/TA2/KR2 groups on the 4th day. B–F) Positive expression ratios of B) PCNA, C) CD31, D) TNF‐α, E) IL‐6, and F) IL‐10 in the control, HA, HA/TA2, and HA/TA2/KR2 groups (*n* = 3), corresponding to (A). G) Immunofluorescence images of iNOS and CD206 in the wound tissues from the control, HA, HA/TA2, and HA/TA2/KR2 groups. *p* < 0.05 (“^*^”) and *p* < 0.01 (“^**^”).

Subsequently, epidermal cell proliferation, vascularization, and anti‐inflammation in the different groups were characterized by immunohistochemical staining of the wound tissues. Proliferating cell nuclear antigen (PCNA) is a type of mark protein in cell nucleus indicating cell proliferation. Based on this principle, we found more proliferating epithelial cells (red arrows) in the HA/TA2/KR2 group than in the other groups, and the number of positive epithelial cells was twice that of the control group (**Figure** **8**Ai,B). The vascularization marker of cluster of differentiation 31 (CD31) was used to reveal the degree of vascularization, and the groups where in the following order: HA/TA2/KR2 > HA/TA2 ≈ HA ≈ control (Figure 8Aii,C). Pro‐inflammatory cytokines such as TNF‐α and IL‐6 showed a downward trend in the HA/TA2/KR2 group, in contrast to the other groups (Figure 8Aiii,iv,D,E). The anti‐inflammatory cytokine IL‐10 showed an observable increase in the HA/TA2/KR2 group (Figure 8Av,F). Moreover, immunofluorescence staining of wound tissues revealed that the M1 type macrophage marker iNOS sharply decreased and the M2 type macrophage marker CD206 significantly increased in the HA/TA2/KR2 group, indicating that the HA/TA2/KR2 cryogel could effectively regulate the phenotype of macrophages in vivo (Figure 8G). Moreover, H&E staining revealed that the inflammatory level of the HA/TA2/KR2 group was the lowest, and the immunohistochemical staining of wound tissues on day 8 also showed similar trend in the expression level of PCNA, CD31, TNF‐α, and IL‐6 to that on day 4 (Figure S15, Supporting Information). H&E staining of the heart, liver, spleen, lung, and kidney demonstrated the excellent biocompatibility of the HA/TA2/KR2 group in vivo (Figure S16, Supporting Information). In summary, the in vivo experiments proved that the HA/TA2/KR2 cryogel could effectively perform antibacterial, antioxidative, anti‐inflammatory, and immune‐regulatory functions.

### 2.8 Pro‐Healing Mechanism of HA/TA2/KR2 Cryogel

**Figure 9:**
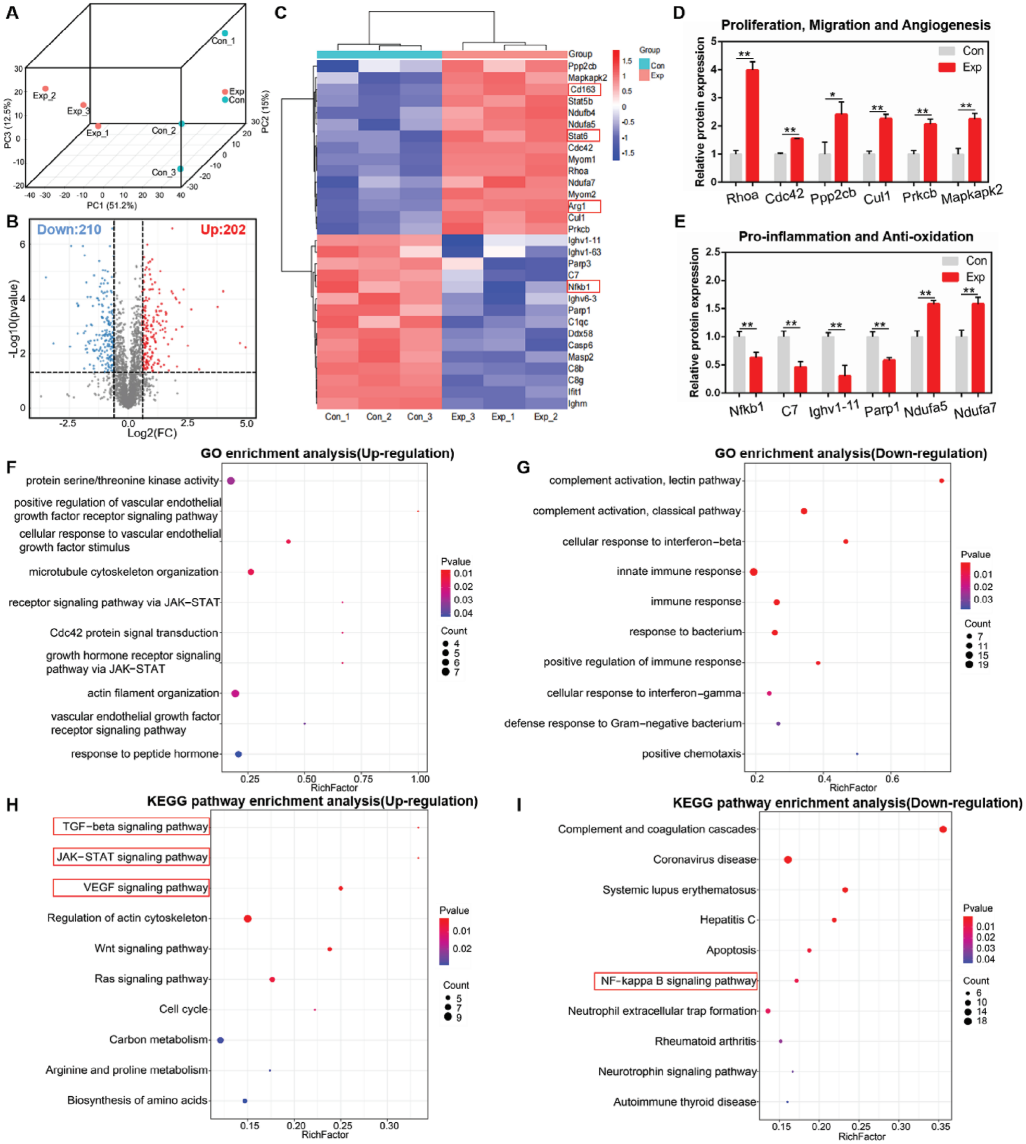
Pro‐healing mechanism of the HA/TA2/KR2 cryogel. A) Unguided 3D principal component analysis (PCA) of differentially expressed proteins in the wound tissues of the control (Con) and HA/TA2/KR2 (Exp) groups (*n* = 3). B) Volcano plot showing upregulated and downregulated proteins after treatment with the HA/TA2/KR2 cryogel. C) Heat map of significant differentially expressed proteins involved in the infected wound microenvironment after treatment with the HA/TA2/KR2 cryogel (fold change ≥ 1.5 and P < 0.05). D,E) Relative expression of proteins related to biological functions, including cell proliferation, migration, angiogenesis, inflammation, and oxidative stress (*n* = 3). F,G) Gene Ontology (GO) enrichment analysis based on upregulated and downregulated proteins in HA/TA2/KR2‐treated wounds versus control wounds. H,I) KEGG pathway enrichment analysis of differentially expressed proteins involved in molecular signaling pathways in HA/TA2/KR2‐treated wounds versus control wounds. *p* < 0.05 (“^*^”) and *p* < 0.01 (“^**^”).

Proteomic analysis was performed to clarify the pro‐healing mechanism of the HA/TA2/KR2 cryogel. As shown in **Figure** **9**A, the principal component analysis (PCA) revealed that protein expression was significantly different between the control and HA/TA2/KR2 groups. A volcano plot revealed that 412 proteins in the two groups changed significantly; 202 and 210 proteins were upregulated and downregulated, respectively (Figure 9B). The heat map shows significant differentially expressed proteins, and we found that markers of M2 type macrophage such as CD163 and Arg1, were highly expressed in the HA/TA2/KR2 group (Figure 9C). Correspondingly, the transcription factor Stat6 that induces macrophage polarization to M2 type was upregulated, while the transcription factor NF‐κb1 that induces macrophage polarization to M1 type was downregulated in the HA/TA2/KR2 group. Based on the relative expression of protein analysis, cell proliferation, migration, and angiogenesis related proteins such as RhoA, Cdc42, Ppp2cb, Cul1, Prkcb, and Mapkapk2 were significantly upregulated in the HA/TA2/KR2 group (Figure 9D). Moreover, compared with the control group, antioxidation‐related proteins such as Ndufa5 and Ndufa7 were also upregulated in the HA/TA2/KR2 group (Figure 9E). In contrast, pro‐inflammatory proteins such as NF‐κb1, C7, Ighv1‐11, and Parp1 were significantly downregulated (Figure 9E). Gene ontology (GO) enrichment analysis revealed that the upregulated proteins focused on the functions of tissue regeneration, such as protein synthesis, cellular response, regulation of VEGF, and cytoskeleton reorganization, while the downregulated proteins focused on inflammatory reactions such as immune response, complement activation, and response to bacteria (Figure 9F,G). Kyoto encyclopedia of genes and genomes (KEGG) pathway analysis indicated that treatment of HA/TA2/KR2 activated the JAK‐STAT6, VEGF, and TGF‐β signaling pathways, and inhibited the NF‐κB signaling pathway (Figure 9H–I). To verify the accuracy of the proteomic analysis, proteins such as NF‐κb1 (p105 and p50), RhoA, Stat6, CD163, and Arg1 were measured by WB. As shown in Figure S17 (Supporting Information), the HA/TA2/KR2 group expressed higher levels of RhoA, Stat6, CD163, and Arg1 than the control group, with lower levels of p105 and p50. These results were consistent with the data presented in the proteomic analysis.

## 3 Discussion

MDR bacteria‐infected wounds, which suffer from poor microenvironments, are the biggest challenge and threat to clinical treatment and public health. As is well known, MDR bacterial colonization in wound tissues not only damages the ECM, but also releases endotoxins (e.g., LPS) or metabolic byproducts that hinder wound healing by inducing a heavy inflammatory response.^[^ ^37^ ^]^ Because of the strong viability of MDR bacteria, the progression of inflammation easily transforms to persistent or chronic inflammation, further causing additional damage to normal tissues.^[^ ^38^ ^]^ In addition, high levels of ROS are other inescapable issues accompanying MDR bacterial infection, inhibiting the proliferation of blood vessels and endothelial cells.^[^ ^39^ ^]^ Therefore, antibacterial, anti‐inflammatory, and anti‐oxidative properties are three key factors for infected wound healing. In this study, we fabricated a multifunctional wound dressing, the HA/TA2/KR2 cryogel, exhibiting excellent abilities, including antibacterial, ROS scavenging, anti‐inflammatory, and macrophage regulation, which completely met the standards of infected wound healing.^[^ ^20^ ^]^ Based on the basic characteristics of MDR bacteria‐infected wounds, we chose the KR‐12 peptide, a broad‐spectrum antimicrobial peptide, as the bactericidal agent, and TA, a natural polyphenol,^[^ ^27^, ^28^ ^]^ to scavenge ROS in wound tissues. In addition, the porous structure endowed the HA/TA2/KR2 cryogel with various advantages such as gas permeability, liquid absorption, flexibility, and shape memory capability. Notably, liquid absorption was beneficial for the absorption of exudates from wounds infected with MDR bacteria. In short, all these characteristics could effectively promote the healing of the MDR bacteria‐infected wounds.

Most studies have only focused on bacterial killing, neglecting the post‐antibacterial period, in which immune cells play important roles. This study focuses on a novel strategy that combines antibacterial and ROS‐scavenging activities with immune regulation during the healing of MDR bacteria‐infected wounds. The experimental results demonstrated that the KR‐12 peptide as a cationic molecule could efficiently kill various bacteria by destroying the cell membranes, reaching a killing ratio of >97% of MDR‐PA bacteria in vivo, proving that HA/TA2/KR2 could perform its antibacterial function in real situations. Additionally, the combination of TA and KR‐12 peptide effectively scavenged ROS in infected wounds. Other characteristics, such as biocompatibility and promotion of HaCat cells (epithelial cell) proliferation and migration, also promote wound healing. More importantly, both the in vitro and in vivo experiments showed that the HA/TA2/KR2 cryogel mediated the polarization of macrophages to suppress inflammation and promote MDR bacteria‐infected wounds. The experimental results showed that our novel strategy was effective, and the pro‐healing capability probably originated from two aspects: 1) the combined effect of antibacterial, anti‐inflammatory, and ROS scavenging, and 2) immune regulation.

Regarding the combined effect, the clearance of bacteria in wound tissues can accelerate the extinction of ROS and further downregulate inflammation.^[33^ ^]^ In addition, inflammation control and ROS scavenging by the combined action of TA and KR‐12 can prevent continuous damage to wound tissues, which in turn destroys the breeding ground of bacteria.^[^ ^5^ ^]^ Combined effect means that the source of inhibiting wound healing can be removed and the microenvironment can be improved. Utilizing KR‐12 and TA to build a gentle microenvironment can be considered a key step in the healing of infected wounds. H&E staining, immunofluorescence staining, and immunohistochemical imaging of wound tissues demonstrated that the synergistic effect of antibacterial, anti‐inflammatory, and ROS‐scavenging activities could improve the wound microenvironment. On the other hand, wound healing involves inflammatory and reparative stages, where macrophages are key immune cells; thus, immune regulation is also important and meaningful for infected wounds.^[^ ^8^, ^12^ ^]^ In vitro experiments, including WB and immunofluorescent staining, showed that HA/TA2/KR2 could stimulate the polarization of macrophages toward the M2 type under an inflammatory microenvironment. Evidence of macrophage phenotype transformation were found when immunofluorescent staining of wound tissues was used, which proved that the regulation of the macrophage phenotype could also work in infected wound molds. When macrophages transform from the M1 to the M2 type, inflammatory cytokines are gradually reduced, while anti‐inflammatory cytokines and reparative factors increase simultaneously, accelerating the healing process of infected wounds. In summary, these characteristics endowed the HA/TA2/KR2 cryogel with an excellent ability to promote MDR bacteria‐infected wound healing.

To further investigate the underlying mechanism of HA/TA2/KR2 cryogel in infected wound healing, we collected wound tissues from the control and HA/TA2/KR2 groups and conducted a proteomic analysis. The data of significant differentially expressed proteins and GO enrichment analysis revealed that HA/TA2/KR2 promoted cell proliferation, migration, and angiogenesis, while decreasing the inflammatory response. In addition, M2 type macrophages’ marker proteins Arg1 and CD163 were upregulated in the HA/TA2/KR2 group. All the changes were consistent with the immunohistochemical and immunofluorescence staining results from the in vivo experiments, further demonstrating the pro‐healing efficacy of the HA/TA2/KR2 cryogel. Notably, we found that the HA/TA2/KR2 treatment inhibited the expression of NF‐κb1 and downregulated the NF‐κB signaling pathway. NF‐κb1 is a crucial transcription factor that modulates inflammatory response and oxidative stress, activating the NF‐κB signaling pathway to induce the polarization of macrophages toward M1 type.^[^ ^40^, ^41^, ^42^ ^]^ Thus, the HA/TA2/KR2 treatment could reduce inflammation and oxidative stress by inhibiting NF‐κB signaling pathway, and promote the polarization of macrophages toward M2 type to an extent. More importantly, we also observed that both the key protein Stat6 and the signaling pathway of JAK‐STAT6 that mediated M2 type macrophages polarization were upregulated in the HA/TA2/KR2 group.^[^ ^39^ ^]^ Therefore, the attenuation of inflammation and the regulation of macrophage phenotype under HA/TA2/KR2 treatment were achieved by suppressing the NF‐κB signaling pathway and stimulating the JAK‐STAT6 signaling pathway. Correspondingly, the TGF‐β and VEGF signaling pathways were also found to be upregulated, further explaining the enhancements in cell proliferation, migration, and angiogenesis after the treatment with HA/TA2/KR2 cryogel.^[^ ^43^, ^44^ ^]^ Taken together, HA/TA2/KR2 cryogel reshaped the infected wound microenvironment, mainly by inhibiting the NF‐κB signaling pathway and activating the JAK‐STAT6 signaling pathway, resulting in rapid angiogenesis and re‐epithelialization, finally promoting the healing of MDR bacteria‐infected wounds.

Because infected wounds caused by MDR bacteria seriously threaten public health and pose a great medical burden to society, researchers have made many efforts to develop new strategies to address this problem. For example, Wu et al. and Zhu et al. designed a dextran‐ and peptide‐based hydrogel and a thermosensitive hydrogel loaded with dipotassium glycyrrhizate, respectively, both of which exhibited excellent antibacterial and anti‐inflammatory activities.^[^ ^45^, ^46^ ^]^ However, the lack of intrinsic antioxidative properties may prevent them from comprehensively improving wound healing efficacy in practice, because high ROS levels in infected wounds can severely hinder tissue regeneration. In contrast, our prepared HA/TA2/KR2 cryogel possessed a prominent ROS‐scavenging capacity both in vitro and in vivo (≈85%, Figures 4E and 7G), which is similar to previous reports.^[^ ^47^, ^48^ ^]^ Combined with its excellent bactericidal and anti‐inflammatory activities, the HA/TA2/KR2 cryogel significantly refined the microenvironment of infected wounds and promoted the healing process, as demonstrated by the in vivo animal results. Luo et al. also developed a multifunctional photothermal hydrogel with antibacterial, anti‐inflammatory, and antioxidative properties to treat infected wounds.^[^ ^49^ ^]^ However, the bacterial efficacy of this dressing was much lower than that of our cryogel (>80% vs >93%), indicating that photothermal hydrogel may not be effective against infected wounds with a heavy bacterial burden. Recently, Miranda‐Calderon et al. reported a wound dressing loaded with ciprofloxacin and rifampicin for the treatment of infected wounds.^[^ ^50^ ^]^ Although this dressing has robust antibacterial and antibiofilm capacities, the potential drug resistance that could result from the long‐term use of antibiotics remains a concern. However, this problem is largely negligible in the treatment of emerging antimicrobial peptides. Collectively, our KR‐12 peptide‐based bactericidal cryogel with its anti‐inflammatory and antioxidative properties is an ideal choice for the treatment of infected wounds.

## 4 Conclusion

A novel antibacterial dressing of HA/TA2/KR2 cryogel was fabricated to deliver the antimicrobial peptide KR‐12 for the healing of MDR bacteria‐infected wounds. The porous structure gives the HA/TA2/KR2 cryogel excellent water absorbance and swelling properties, further demonstrating its flexibility, elasticity, and shape memory capability. The mechanical performance of the HA/TA2/KR2 cryogel revealed its better hemostasis capability than that of commercial materials, such as gauze and Alginate Ag. Because HA can be decomposed by the hyaluronidase produced by bacteria, KR‐12 in the cryogel can be released responsively to kill bacteria in infected wounds. The HA/TA2/KR2 cryogel not only exhibited broad‐spectrum antibacterial activity with a clear ratio > 93%, but also downregulated inflammation and altered the macrophage phenotype. More importantly, ROS scavenging by TA could effectively block the sources of damage to normal wound tissues and cooperate with KR‐12 to prevent all obstacles to wound healing. The proteomic analysis proved that the HA/TA2/KR2 cryogel mainly inhibited the NF‐κB and stimulated the JAK‐STAT6 signaling pathway to achieve anti‐inflammatory and antioxidative properties and to promote polarization of macrophages to M2 type. In summary, the HA/TA2/KR2 cryogel not only efficiently delivered the antimicrobial peptide KR‐12 but also exhibited antioxidative and anti‐inflammatory functions in vivo and in vitro. Hence, antioxidation, anti‐inflammation, and immune regulation‐integrated antibacterial cryogels are being developed for infected wound dressings.

## 5 Experimental Section

The details are shown in the Supporting Information.

## Conflict of Interest

The authors declare no conflict of interest.

## Supporting information

Supporting Information

## Data Availability

The data that support the findings of this study are available from the corresponding author upon reasonable request.

## References

[1] Y. Qian , S. Deng , Z. Cong , H. Zhang , Z. Lu , N. Shao , S. A. Bhatti , C. Zhou , J. Cheng , S. H. Gellman , R. Liu , J. Am. Chem. Soc. 2022, 144, 1690. 35007085 10.1021/jacs.1c10659

[2] Y. Zhu , C. Shao , G. Li , Z. Lai , P. Tan , Q. Jian , B. Cheng , A. Shan , J. Med. Chem. 2020, 63, 9421. 32706256 10.1021/acs.jmedchem.0c00583

[3] World Health Organization fact sheet – antibiotic resistance , https://www.who.int/news‐room/fact‐sheets/detail/antibiotic‐resistance (accessed: November 2018).

[4] C. Dunnill , T. Patton , J. Brennan , J. Barrett , M. Dryden , J. Cooke , D. Leaper , N. T. Georgopoulos , Int. Wound J. 2017, 14, 89. 26688157 10.1111/iwj.12557 PMC7950185

[5] Z. Xu , S. Han , Z. Gu , J. Wu , Adv. Healthcare Mater. 2020, 9, 1901502. 10.1002/adhm.201901502 31977162

[6] C. C. Winterbourn , Nat. Chem. Biol. 2008, 4, 278. 18421291 10.1038/nchembio.85

[7] P. P. Kalelkar , M. Riddick , García , Nat. Rev. Mater. 2022, 7, 39. 35330939 10.1038/s41578-021-00362-4 PMC8938918

[8] M. Kharaziha , A. Baidya , N. Annabi , Adv. Mater. 2021, 33, 2100176. 10.1002/adma.202100176 PMC8489436 34251690

[9] P. J. Murray , J. E. Allen , S. K. Biswas , E. A. Fisher , D. W. Gilroy , S. Goerdt , S. Gordon , J. A. Hamilton , L. B. Ivashkiv , T. Lawrence , M. Locati , A. Mantovani , F. O. Martinez , J. L. Mege , D. M. Mosser , G. Natoli , J. P. Saeij , J. L. Schultze , K. A. Shirey , A. Sica , J. Suttles , I. Udalova , J. A. van Ginderachter , S. N. Vogel , T. A. Wynn , Immunity. 2014, 41, 14. 25035950 10.1016/j.immuni.2014.06.008 PMC4123412

[10] C. Cai , X. Zhang , Y. Li , X. Liu , S. Wang , M. Lu , X. Yan , L. Deng , S. Liu , F. Wang , C. Fan , Adv. Mater. 2022, 34, 2106564. 10.1002/adma.202106564 34816470

[11] S. Liu , Q. Zhang , J. Yu , N. Shao , H. Lu , J. Guo , X. Qiu , D. Zhou , Y. Huang , Adv. Healthcare Mater. 2020, 9, 2000198. 10.1002/adhm.202000198 32338465

[12] Z. Tu , M. Chen , M. Wang , Z. Shao , X. Jiang , K. Wang , Z. Yao , S. Yang , X. Zhang , W. Gao , C. Lin , B. Lei , C. Mao , Adv. Funct. Mater. 2021, 31, 2100924.

[13] Y. Wang , Y. Yang , Y. Shi , H. Song , C. Yu , Adv. Mater. 2020, 32, 1904106. 10.1002/adma.201904106 31799752

[14] G. Li , Z. Lai , A. Shan , Adv. Sci. 2023, 10, 2206602. 10.1002/advs.202206602 PMC10104676 36722732

[15] J. Lojk , J. Repas , P. Veranič , V. B. Bregar , M. Pavlin , Toxicology. 2020, 432, 152364. 31927068 10.1016/j.tox.2020.152364

[16] M. Enea , E. Pereira , M. P. de Almeida , A. M. Araújo , M. de Lourdes Bastos , H. Carmo , Nanomaterials. 2020, 10, 995. 32455923 10.3390/nano10050995 PMC7279525

[17] Y. Q. Zhao , Y. Sun , Y. Zhang , X. Ding , N. Zhao , B. Yu , H. Zhao , S. Duan , F. J. Xu , ACS Nano. 2020, 14, 2265. 32017535 10.1021/acsnano.9b09282

[18] P. Tan , H. Fu , X. Ma , Nano Today. 2021, 39, 101229.

[19] S. Wu , Y. Yang , S. Wang , C. Dong , X. Zhang , R. Zhang , L. Yang , Carbohydr. Polym. 2022, 278, 118994. 34973798 10.1016/j.carbpol.2021.118994

[20] M. Liu , T. Liu , X. Zhang , Z. Jian , H. Xia , J. Yang , X. Hu , M. Xing , G. Luo , J. Wu , Int. J. Nanomed. 2019, 14, 3345. 10.2147/IJN.S199618 PMC6516050 31190796

[21] D. W. Song , S. H. Kim , H. H. Kim , K. H. Lee , C. S. Ki , Y. H. Park , Acta Biomater. 2016, 39, 146. 27163404 10.1016/j.actbio.2016.05.008

[22] X. Zhao , B. Guo , H. Wu , Y. Liang , P. X. Ma , Nat. Commun. 2018, 9, 2784. 30018305 10.1038/s41467-018-04998-9 PMC6050275

[23] Y. Yu , P. Li , C. Zhu , N. Ning , S. Zhang , G. J. Vancso , Adv. Funct. Mater. 2019, 29, 1904402.

[24] L. Han , P. Li , P. Tang , X. Wang , T. Zhou , K. Wang , F. Ren , T. Guo , X. Lu , Nanoscale. 2019, 11, 15846. 31289795 10.1039/c9nr03095f

[25] H. Y. Lee , C. H. Hwang , H. E. Kim , S. H. Jeong , Carbohydr. Polym. 2018, 186, 290. 29455990 10.1016/j.carbpol.2018.01.056

[26] R. Stern , G. Kogan , M. J. Jedrzejas , L. Soltés , Biotechnol. Adv. 2007, 25, 537. 17716848 10.1016/j.biotechadv.2007.07.001

[27] N. Xu , Y. Yuan , L. Ding , J. Li , J. Jia , Z. Li , D. He , Y. Yu , Burns Trauma. 2022, 10, tkac019. 35910193 10.1093/burnst/tkac019 PMC9327735

[28] N. Xu , Y. Gao , Z. Li , Y. Chen , M. Liu , J. Jia , R. Zeng , G. Luo , J. Li , Y. Yu , Chem. Eng. J. 2023, 466, 143173.

[29] J. Zhang , M. M. Wu , P. Peng , J. Q. Liu , J. Lu , S. X. Qian , J. Feng , ACS Appl. Mater. Interfaces. 2022, 14, 56097. 36484598 10.1021/acsami.2c17272

[30] L. H. Yuwen , Q. Qiu , W. J. Xiu , K. L. Yang , Y. Q. Li , H. Xiao , W. J. Yang , D. L. Yang , L. H. Wang , Biomater. Sci. 2021, 9, 4484. 34002742 10.1039/d1bm00406a

[31] O. Ciofu , C. Moser , P. Jensen , N. Høiby , Nat. Rev. Microbiol. 2022, 20, 621. 35115704 10.1038/s41579-022-00682-4

[32] S. B. Nimse , D. Pal , RSC Adv. 2015, 5, 27986.

[33] L. Turell , A. Zeida , M. Trujillo , Essays Biochem. 2020, 64, 55. 31919496 10.1042/EBC20190053

[34] S. B. Xu , L. N. Chang , Y. A. Hu , X. J. Zhao , S. C. Huang , Z. H. Chen , X. L. Ren , X. F. Mei , J. Nanobiotechnol. 2021, 19, 362. 10.1186/s12951-021-01106-w PMC8579683 34758829

[35] L. S. Zhou , L. M. Zhou , C. X. Wei , R. Guo , Carbohydr. Polym. 2022, 291, 119558. 35698384 10.1016/j.carbpol.2022.119558

[36] A. Hassanshahi , M. Moradzad , S. Ghalamkari , M. Fadaei , A. J. Cowin , M. Hassanshahi , Cells. 2022, 11, 2953. 36230913 10.3390/cells11192953 PMC9564023

[37] M. D. Caldwell , Surg. Clin. North Am. 2020, 100, 757. 32681875 10.1016/j.suc.2020.05.007

[38] L. Chen , H. Deng , H. Cui , J. Fang , Z. Zuo , J. Deng , Y. Li , X. Wang , L. Zhao , Oncotarget. 2018, 9, 7204. 29467962 10.18632/oncotarget.23208 PMC5805548

[39] H. Zhao , J. Huang , Y. Li , X. Lv , H. Zhou , H. Wang , Y. Xu , C. Wang , J. Wang , Z. Liu , Biomaterials. 2020, 258, 120286. 32798744 10.1016/j.biomaterials.2020.120286

[40] B. George , T. V. Suchithra , N. Bhatia , Inflammation Res. 2021, 70, 51. 10.1007/s00011-020-01426-x 33245371

[41] F. Cai , W. Chen , R. Zhao , Y. Liu , Mol. Biol. Rep. 2023, 50, 5355. 37029875 10.1007/s11033-023-08392-7

[42] C. Yunna , H. Mengru , W. Lei , C. Weidong , Eur. J. Pharmacol. 2020, 877, 173090. 32234529 10.1016/j.ejphar.2020.173090

[43] S. Chattopadhyay , L. B. C. Teixeira , L. L. Kiessling , J. F. McAnulty , R. T. Raines , ACS Chem. Biol. 2022, 17, 314. 35084170 10.1021/acschembio.1c00745 PMC8857044

[44] S. P. Herbert , D. Y. Stainier , Nat. Rev. Mol. Cell Biol. 2011, 12, 551. 21860391 10.1038/nrm3176 PMC3319719

[45] S. W. Wu , Y. L. Yang , S. A. Wang , C. Y. Dong , X. Y. Zhang , R. Zhang , L. Yang , Carbohydr. Polym. 2022, 278, 118994. 34973798 10.1016/j.carbpol.2021.118994

[46] D. Y. Zhu , Z. P. Chen , Z. P. Hong , L. Y. Zhang , X. X. Liang , Y. Li , X. J. Duan , H. S. Luo , J. P. Peng , J. W. Guo , Acta Biomater. 2022, 143, 203. 35245682 10.1016/j.actbio.2022.02.041

[47] C. X. Tu , H. D. Lu , T. Zhou , W. Y. Zhang , L. W. Deng , W. B. Cao , Z. J. Yang , Z. L. Wang , X. Y. Wu , J. Ding , F. Xu , C. Y. Gao , Biomaterials. 2022, 286, 121597. 35688112 10.1016/j.biomaterials.2022.121597

[48] X. J. Yu , X. X. Fu , J. X. Yang , L. Chen , F. Leng , Z. Y. Yang , C. Yu , Mater. Today Bio. 2022, 15, 100308. 10.1016/j.mtbio.2022.100308 PMC9194460 35711291

[49] Y. D. Luo , X. D. Zhou , C. K. Liu , R. L. Lu , M. Q. Jia , P. F. Li , S. Y. Zhang , Biomater. Adv. 2022, 141, 213096. 36067644 10.1016/j.bioadv.2022.213096

[50] L. Miranda‐Calderon , C. Yus , C. R. de Ganuza , M. Paesa , G. Landa , E. Tapia , E. Perez , M. Pérez , V. Sebastian , S. Irusta , G. Mendoza , M. Arruebo , Chem. Eng. J. 2023, 476, 146679.

