# Supplementary material for: Hyaluronidase‐Responsive Bactericidal Cryogel for Promoting Healing of Infected Wounds: Inflammatory Attenuation, ROS Scavenging, and Immune Regulation
Source: Adv Sci (Weinh). 2024 Feb 13;11(17):2306602. doi: 10.1002/advs.202306602 (PMC11077649; doi:10.1002/advs.202306602)
Supplement: Supplementary file 1 — Supporting Information [file ADVS-11-2306602-s001.pdf]

ADVANCED SCIENCE
Open Access

Supporting Information

for *Adv. Sci.*, DOI 10.1002/advs.202306602

Hyaluronidase-Responsive Bactericidal Cryogel for Promoting Healing of Infected Wounds: Inflammatory Attenuation, ROS Scavenging, and Immune Regulation

Menglong Liu, Rui Ding, Zheng Li, Na Xu, Yali Gong, Yong Huang, Jiezhi Jia, Haiyan Du, Yunlong Yu* and Gaoxing Luo**

Supporting Information

Hyaluronidase-Responsive Bactericidal Cryogel For Promoting Healing Of Infected Wounds: Inflammatory Attenuation, ROS Scavenging, And Immune Regulation

Menglong Liu[1#], Rui Ding[2#], Zheng Li[1#], Na Xu[1], Yali Gong[1], Yong Huang[1], Jiezhi Jia[1], Haiyan Du[2*], Yunlong Yu[1*], Gaoxing Luo[1*]

[1]Institute of Burn Research, State Key Laboratory of Trauma, Burns and Combined Injury, Southwest Hospital, Third Military Medical University (Army Medical University), Gaotanyan Street, Shapingba District, Chongqing 400038, China.

[2]College of Chemical Engineering and Technology, Taiyuan University of Technology, Yingze West Street 79, Taiyuan 030024, China.

[#]Contribute equally to this paper

[*]**Correspondence:**

Prof. Gaoxing Luo

Institute of Burn Research, State Key Laboratory of Trauma, Burns and Combined Injury, Southwest Hospital, Third Military Medical University (Army Medical University), Gaotanyan Street, Shapingba District, Chongqing 400038, China

Tel:+86-023-68754173

E-mail: Gaoxing.luo@burnstrauma.com

Prof. Yunlong Yu

Institute of Burn Research, State Key Laboratory of Trauma, Burns and Combined Injury, Southwest Hospital, Third Military Medical University (Army Medical University), Gaotanyan Street, Shapingba District, Chongqing 400038, China

E-mail: yuyunlong666@gmail.com

Prof. Haiyan Du

College of Chemical Engineering and Technology, Taiyuan University of Technology, Yingze West Street 79, 030024 Taiyuan, China

E-mail: duhaiyan428@163.com

ORCID ID: 0000-0002-2526-4374 (Menglong Liu)

Experimental Section

1. Materials and animals

Sodium hyaluronate (HA, Mw: 1.3-1.4 MDa) was purchased from Bloomage Biotechnology Co., Ltd. (Shandong, China). Tannic acid (TA, Mw: 1701.2 Da) and polyethylene glycol diglycidyl ether (PEGDE, Mw: 500 Da) were purchased from Sigma-Aldrich (Milwaukee, WI, USA). KR-12 peptide (CKRIVKRIKKWLR, >98% purity) was obtained from Sangon (Shanghai, China). A Cysteine residue was introduced at the N-terminus of the KR-12 peptide to make it possess an intrinsic anti-oxidation effect and ease of modification[1]. Methylene blue (MB, Mw: 373.9 Da) and hyaluronidase (HAase, Mw: 55 KDa) were purchased from Solarbio Science & Technology Co., Ltd. (Beijing, China). The methicillin-resistant *Staphylococcus aureus* (MRSA, ATCC 43300), multi-drug-resistant bacterial strain of *Pseudomonas aeruginosa* (MDR-PA, 202106023) and non-drug-resistant bacterial strain of *Escherichia coli* (*E. coli*, ATCC 25923) were obtained from the Clinical Microbiology Laboratory, Institute of Burn Research, Southwest Hospital, Army Medical University (AMU, Chongqing, China).

BALB/c mice (male, 18-20 g, 6-8 weeks old), C57BL/6 mice (male, 18-20 g, 6-8 weeks old) and SD rats (male, 200-250 g, 7-8 weeks old) were purchased from the Animal Department of Army Medical University (AMU, Chongqing, China). All the animal experimental methods were approved by the ethical committee of AMU (AMUWEC20210410, AMUWEC20211786). All the animal experiments were performed in accordance with the guidelines of AMU.

2. Preparation of HA/TA/KR cryogels

Briefly, HA was dissolved in deionized water at 60 °C in a water bath to form a 2% (w/v) solution according to previous studies[2-3]. To crosslink HA, 0.5% (v/v) PEGDE was added to the HA solution and stirred at 25 °C for 12 h. TA was then added to the solution at different concentrations (0.1%, 0.2%, and 0.5% [w/v]), and the specimens were denoted as HA/TA1, HA/TA2, and HA/TA5, respectively. After that, KR-12 peptide solution was dripped into the HA/TA2 solution at different concentrations (1, 2, and 4 mg/mL) and stirred at 25 °C for 12 h. Finally, the mixture solution was added into a mold, and the mold was placed in a freezer at

−80 °C for 24 h to form cryogels. These specimens were denoted as HA/TA2/KR1, HA/TA2/KR2, and HA/TA2/KR4, respectively. The cryogel formed by the KR-12 peptide (2 mg/mL) mixed with HA solution (2% [w/v]) was denoted as HA/KR2, and the cryogels formed by the KR-12 peptide (2 mg/mL) mixed with HA/TA1 and HA/TA5 solution were denoted as HA/TA1/KR2 and HA/TA5/KR2. All specimens were sterilized under UV light prior to the subsequent biological experiments.

3. Characterization of cryogels

The chemical characteristics of the cryogels were investigated using a Fourier transform-infrared (FTIR) spectrometer (Thermo Scientific Nicolet iS20, USA) in the range of 4000−400 cm^{-1}. The interior morphology of the cryogels was observed using a scanning electron microscopy (SEM, Crossbeam 340, Zeiss, Germany). Based on the SEM images, the pore diameters of the cryogels were measured using ImageJ software. The mechanical properties of the cryogels were investigated by compression testing using a universal mechanical tester (Exceed 44, Germany). Briefly, cryogels were prepared with a diameter of 8 mm and a height of 10 mm. A maximum compressive strain of 80% was selected for testing at a speed of 0.1 mm/s. Before the compression test, a drop of deionized water was added to the samples on the platform. To detect shape memory ability, a cryogel shaped like a five-pointed star was first obtained by freeze-drying using a specific mold. After strong compression, the cryogel was immersed in deionized water for 15 s and photographs were recorded. To detect flexibility, a cryogel was severely bent, twisted, and compressed after absorbing water, and photographs were recorded.

4. Porosity, swelling ratio and in vitro degradation

The porosity of the cryogels was investigated using the liquid displacement method. Briefly, pre-weighed cryogels were immersed in absolute ethanol under ultrasonication for 10 min. After removing the excess ethanol, the samples were weighed again. The percentage porosity was calculated using the following equation:

$$\text{Porosity (\%)} = (m_1 - m_0)/(v \times \rho) \times 100\%$$

where m_0 and m_1 represent the weights of cryogels before and after immersing in absolute ethanol, respectively, v represents the volume of cryogels, and ρ represents the density of

absolute ethanol.

The swelling ratio was evaluated by measuring the mass of the cryogel before and after immersion in a PBS solution (pH 7.4). First, dried cryogels were weighed. After that, the cryogels were immersed in PBS at 37 °C for 30 min. The cryogels were then weighed again after removing the excess solution. The swelling ratios were calculated as follows:

$$\text{Swelling ratio (\%)} = (W_1 - W_0)/W_0 \times 100\%$$

where W_0 and W_1 represent the weights of the cryogels before and after immersion in PBS, respectively.

In vitro degradation properties were tested by calculating the weight loss of the cryogels after immersion in PBS with or without hyaluronidase (100 U/mL) for different time periods.[4-5]. At first, the pre-weighed cryogels were immersed in the above solution at 37 ℃. Subsequently, the cryogels were removed from the solution at predetermined time points and weighed again after freeze-drying. Weight loss was calculated as follows:

$$\text{Weight loss (\%)} = (M_n - M_0)/M_0 \times 100\%$$

where M_0 represents the weight of the cryogel before immersion in solution, and M_n represents the weight of the cryogel after immersion in solution at predetermined time.

5. Hyaluronidase measurement

To measure the hyaluronidase (HAase) produced by bacteria, a fluorescence-based test was conducted using the methylene blue (MB) as previously described[6]. In brief, hyaluronidase was serially diluted in different concentrations with PBS (0, 25, 50, 100, and 200 U/mL). Then HA at a final concentration of 0.05 mg/mL and MB at a final concentration of 4 μM were added to the hyaluronidase, and the mixture solution was incubated for 1 h at 37 °C. Afterward, the optical density was measured at 680 nm using a microplate reader (Thermo Scientific Varioskan Flash, USA), and a standard curve was drawn with these data. MDR-PA bacteria (1×10^6 CFU) were added in 5 mL of LB media and incubated overnight for 24 h in a 37 ℃ shaker to obtain the sample. As mentioned above, 100 μL of bacterial cell lysate and supernatant were incubated with HA and MB for 1 h at 37 °C, and the optical density was measured. Finally, the concentration of hyaluronidase produced by bacteria was calculated using the standard curve. The data was 93.4 U/mL, which was close to the

concentration used for in vitro experiments (100U/mL) mentioned in previous studies. Therefore, we select three different concentrations of HAase (50, 100, and 200 U/mL) for the following evaluation of the release sensitivity of KR-12 peptide after HAase action.

6. In vitro cell proliferation and migration assay

HaCat and NIH3T3 cells were incubated in RPMI1640 medium (Gibco, USA) containing 10% fetal bovine serum (FBS; HyClone, USA) and Dulbecco's modified Eagle's medium (DMEM; Gibco, USA) containing 10% FBS at 37 °C in a 5% CO2 incubator. For the cell proliferation assay, the cells were seeded in a 24-well plate at a concentration of 2 × 104 cells/well. Subsequently, 3.2 mg (freeze-dried from 100 μL solution) of HA, HA/TA2, HA/TA2/KR1, HA/TA2/KR2, and HA/TA2/KR4 cryogels were placed in transwell inserts for co-incubation with cells. Cells treated with only a blank transwell insert served as a control group. On days 1, 3, 5, and 7, cell proliferation was measured using Cell Counting Kit-8 (CCK-8, Solarbio Science & Technology, China), and the optical density (OD) values in each group were measured at 450 nm using a microplate reader (Thermo Scientific Varioskan Flash, USA). Cell viability was calculated using the following equation:

$$\text{Cell viability (\%)} = [(OD_{sample} – OD_{medium})/(OD_{control} – OD_{medium})] \times 100\%$$

where OD_{sample} represents the OD values of the HA, HA/TA2, HA/TA2/KR1, HA/TA2/KR2, and HA/TA2/KR4 groups; OD_{medium} represents the OD value of the pure medium; and $OD_{control}$ represents the OD value of the control group.

The cytotoxicity of free TA (50, 100, and 200 μg) and KR-12 peptide (50, 100, and 200 μg) on HaCat cells and NIH3T3 cells, and the cytotoxicity of HA/TA1, HA/TA2, and HA/TA5 cryogels on NIH3T3 cells were also detected using the CCK-8 test as mentioned above.

Furthermore, a live/dead staining test was used to assess cell viability on day 3 using a kit (Beyotime Biotechnology, China). Under a fluorescence microscope (Olympus, Japan), living cells stained with calcein-AM showed a green signal, while dead cells stained with propidium iodide (PI) showed a red signal.

For the cell migration assay, 3×10^4 HaCat cells/well were seeded in 24-well plates and grown to confluence. First, a scratch was carefully made at the bottom of each well and the

free cells were gently washed away with PBS. Then, pure fresh medium without FBS was added to each well and 3.2 mg of the HA, HA/TA2, and HA/TA2/KR2 cryogels were placed in transwell inserts for co-incubation with the cells. Cells treated with only a blank transwell insert served as a control group. The cells were imaged using a fluorescence microscope (Olympus, Tokyo, Japan) at 0 and 24 h, and the scratch width was measured using ImageJ software. The migration rate (%) was calculated as follows:

$$\text{Migration rate (\%)} = [(L_0 - L_{24})/L_0] \times 100\%$$

where L_0 represents the initial width and L_{24} represents the final width after co-incubation for 24 h.

7. Hemolysis assay

The anticoagulated rat blood was centrifuged at 1000 rpm for 10 min to obtain red blood cells (RBCs). The RBCs were then washed thrice with PBS and diluted with PBS to a final concentration of 5% (v/v). After that, 500 uL of diluted RBCs was incubated with 3.2 mg of HA, HA/TA2, HA/TA2/KR1, HA/TA2/KR2, and HA/TA2/KR4 cryogels. RBCs incubated with deionized water and saline served as the positive and negative controls, respectively. After incubation for 1 h at 37 °C in a shaker, the RBCs in each group were centrifuged at 1000 rpm for 5 min. The absorbance of the supernatant from each group was measured at 545 nm using a microplate reader. The hemolysis ratio was calculated using the following equation:

$$\text{Hemolysis ratio (\%)} = [(A_{sample} - A_{saline})/(A_{water} - A_{saline})] \times 100\%$$

where A_{sample} represents the absorbance of the HA, HA/TA2, HA/TA2/KR1, HA/TA2/KR2, and HA/TA2/KR4 groups; A_{saline} represents the absorbance of the saline group; and A_{water} represents the absorbance of the deionized water group.

8. In vitro detection of release characteristics of TA and KR-12

To detect the HAase-responsive release characteristics of TA and KR-12, the HA/TA2/KR2 cryogels were incubated with PBS and different concentrations of HAase solution (50, 100, and 200 U/mL) in a shaker at 37 °C, respectively. After incubation for 1h, 6h, 24h, 48h, 72h, and 96h, the supernatants in each group were harvested for measurement.

For the detection of TA, the supernatants were quantified using UV−Vis spectroscopy at

274 nm as previously described[7]. Then the concentration of TA was calculated based on a calibration plot prepared using the same method with the known concentration of TA. The release percentage of TA was calculated using the equation:

$$\text{Release percentage (\%)} = (C \times V)/M \times 100\%$$

where C represents the concentration of TA, V represents the volume of hyaluronidase solution, and M represents the total mass of TA in the cryogel.

For the detection of KR-12, an Ellman's assay was used as previously described[8]. Briefly, the supernatants were incubated with 5, 5′-dithiobis-(2-nitrobenzoic acid) (DTNB, Aladdin Biochemical Technology Co., Ltd., China), and the absorbance value of the solution was measured at 412 nm by a microplate reader (Thermo Scientific Varioskan Flash, USA). Then the concentration of KR-12 was calculated based on a calibration plot prepared using the same method with the known concentration of KR-12. The release percentage of KR-12 was calculated using the equation:

$$\text{Release percentage (\%)} = (C \times V)/M \times 100\%$$

where C represents the concentration of KR-12, V represents the volume of hyaluronidase solution, and M represented the total mass of KR-12 in the cryogel.

To detect the effect of TA binding on the release of KR-12, cryogels containing different concentrations of TA (0, 0.1, 0.2, and 0.5% [w/v]), represented as HA/KR2, HA/TA1/KR2, HA/TA2/KR2, and HA/TA5/KR2, respectively, were incubated with HAase (100 U/mL) in a shaker at 37 ℃. After incubation for 1 h, 6 h, 24 h, 48 h, 72 h, and 96 h, the supernatants in each group were harvested for measurement using an Ellman's assay as mentioned above.

The bacteria-responsive releasing characteristic of the HA/TA2/KR2 cryogel was also evaluated. Briefly, the cryogels were incubated with PBS and bacterial suspension of MDR-PA (5×10^6 CFU/mL) in a shaker at 37 ℃, respectively. Then, the supernatants were harvested and detected using an Ellman's assay as mentioned above.

9. In vitro antibacterial assay

The antibacterial properties of the cryogels were evaluated against gram-positive MRSA and gram-negative *E. coli* and MDR-PA. Briefly, a bacterial suspension at a concentration of 5×10^6 CFU/mL was first obtained by diluting the log-phase bacterial solution with PBS (pH

7.4). Then, 400 μL of diluted bacterial suspension and 3.2 mg of cryogel were added into the 24-well plate, and the mixture solution was incubated for 6 h at 37 ℃ in a shaker. The groups were divided into control (pure bacterial suspension), HA, HA/TA2, HA/TA2/KR1, HA/TA2/KR2, and HA/TA2/KR4. For standard plate counting method, the suspension was harvested and diluted with PBS, and 20 μL of final solution was uniformly spread on an agar plate. After incubation for 24 h at 37 °C, the agar plate was imaged, and the number of bacterial colonies was counted using an automatic colony counter (Supcre, Shineso, China). For the live/dead staining assay, the bacterial suspensions were separately harvested after incubation for 2 and 6 h and stained using a Live/Dead™ Baclight™ Bacterial Viability Kit (Invitrogen, USA), according to the manufacture's instruction. The stained bacteria were observed under a fluorescence microscope (Olympus, Japan); green fluorescence represented living bacteria, while red fluorescence represented dead bacteria. For SEM observations, the bacterial suspensions were harvested after incubation for 2 and 6 h and dripped onto glass slides. After drying, the samples were fixed in 4% formaldehyde, dehydrated using a series of ethanol solutions, and sprayed with a gold layer. Finally, the micromorphology of the bacteria was observed using SEM (CrossBeam 340, Zeiss, Germany). For the measurement of leakage protein content from broken bacteria, the supernatants were harvested, and the total protein concentration was measured using a BCA protein test kit according to the manufacturer's instructions (Beyotime, China).

To explore the long-term antibacterial activity of HA/TA2/KR2 cryogel and free KR-12 peptide, a bacterial suspension assay was adopted as previously described[8]. Briefly, 3.2 mg of cryogel and 200 μg of free KR-12 (equivalent mass to the KR-12 in cryogel) were incubated with MDR-PA bacterial cells (5×10^6 CFU/mL in LB medium) in a shaker at 4 ℃. The bacterial suspension without any treatment served as a control group. After incubation for 1 day, 3 days, 5 days, and 7 days, the number of bacteria was measured using a standard plate counting method as mentioned above.

To further investigate the anti-biofilm efficacy of the cryogels, a bacterial suspension of MDR-PA was first diluted with tryptic soy broth (TSB, Solarbio Science & Technology, China) to a concentration of 5×10^6 CFU/mL. Then, 400 μL of diluted bacterial suspension

and 3.2 mg of cryogel were added onto a pre-placed glass slide in the well of a 24-well plate, and the mixture solution was incubated overnight at 37 ℃ in a shaker. Afterwards, the 24-well plate was incubated at 37 ℃ for 48 h without interference to form biofilm. The bacterial biofilm was stained using a Live/Dead™ Baclight™ Bacterial Viability Kit as described above and analyzed using a confocal laser scanning microscope (CLSM, Olympus, Japan). Bacterial biofilms were stained with 0.5% crystal violet and imaged using a fluorescence microscope (Olympus, Japan). To calculate the anti-biofilm efficacy, the biofilm stained with crystal violet was dissolved in absolute ethanol, and the absorbance of the supernatant was measured at 595 nm using a microplate reader. The antibiofilm efficacy was calculated using the following equation:

$$\text{Anti-biofilm efficacy (\%)} = (A_{control} - A_{sample})/A_{control} \times 100\%$$

where $A_{control}$ represents the absorbance of the control group and A_{sample} represents the absorbance of the HA, HA/TA2, and HA/TA2/KR2 groups.

10. In vitro antioxidation assay

The 1,1-diphenyl-2-picrylhydrazyl (DPPH•, Macklin, China) and 2-phenyl-4,4,5,5-tetramethylimidazoline-3-oxide-1-oxyl (PTIO•, Aladdin, China) free radical scavenging assays were performed according to the manufacturer’s instructions and a previous report[9]. Briefly, 3.2 mg of cryogel powder and 200 μg of free TA (equivalent mass to the TA in cryogel) were uniformly co-incubated with DPPH• or PTIO• in the dark for 60 min, respectively. Free radicals co-incubated with PBS served as the control group. After centrifugation, the absorbances of the DPPH• and PTIO• supernatants were measured at 517 and 557 nm, respectively, using a microplate reader. The scavenging ability was calculated using the following equation:

$$\text{Scavenging effect (\%)} = (A_{control} - A_{sample})/A_{control} \times 100\%$$

where $A_{control}$ represents the absorbance of the control group and A_{sample} represents the absorbance of the HA, HA/TA2, and HA/TA2/KR2 groups.

The intracellular ROS scavenging properties were evaluated using an ROS assay kit according to the manufacturer’s instructions (Beyotime Biotechnology, China). Bone marrow-derived macrophages (BMDMs) used were isolated from C57BL/6 mice and cultured

in RPMI 1640 medium containing 10% FBS and 20 ng/mL M-CSF as previously described[10]. BMDMs were seeded in a 6-well plate at a concentration of 5 × 10^5 cells/well, and then treated with fresh medium containing H_2O_2 (0.5 mM) and co-incubated with 12.8 mg of cryogels placed in the transwell inserts at 37 ℃. Cells treated with pure medium served as the negative control group, whereas cells treated with medium containing H_2O_2 served as the positive control group. Cells treated with 800 μg of free TA served as the TA group. After incubation for 6 h, the medium was removed, and the cells were stained with DCFH-DA probe and Hoechst dye (Beyotime Biotechnology, China) according to the manufacturer's instructions. The cells were observed and imaged using a CLSM (Olympus, Japan). Green fluorescence indicates ROS, and blue fluorescence indicates cell nuclei. The stained cells were harvested to measure the fluorescence intensity using flow cytometry, and the data were analyzed using FlowJo software (Tree Star Incorporation, USA).

To further evaluate the synergistic effect of KR-12 and TA on ROS scavenging in cells, a ROS fluorescence measurement assay was used as mentioned above. There were four groups including Control, HA/TA2, HA/KR2, and HA/TA2/KR2[11-12]. The treatment of HA/TA2 and HA/KR2 represented the single action of TA and KR-12, respectively, and the treatment of HA/TA2/KR2 represented the synergistic action. All data were standardized with data from the control group.

11. Macrophage polarization and anti-inflammation assessment

BMDMs at a concentration of 5 × 10^5 cells/well were seeded on a pre-placed glass slide in the 6-well plate, and stimulated with LPS (200 ng/mL) and IFN-γ (20 ng/mL) for 24 h to induce polarization towards M1 type. Subsequently, 12.8 mg of HA, HA/TA2, and HA/TA2/KR2 cryogels were placed in transwell inserts and co-incubated with the cells for another 24 h. LPS-stimulated BMDMs without any other treatment served as the control group.

For immunofluorescence staining, the cells were fixed in 4% paraformaldehyde for 30 min, followed by three washes with PBS. The cells were then incubated with rabbit polyclonal antibody against CD206 (1:500, Abcam) and mouse monoclonal antibody against CD86 (1:500, Santa cruze) overnight at 4 ℃. After washing with PBS thrice, the cells were

incubated with Cy3 labeled goat anti-rabbit IgG secondary antibody (1:800, Beyotime Biotechnology) and FITC labeled goat anti-mouse IgG secondary antibody (1:800, Beyotime Biotechnology) for 1 h at 37 °C. The cells were washed with PBS and counterstained with 4',6-diamidino-2-phenylindole (DAPI, Beyotime Biotechnology) dye. Finally, the cells were imaged and analyzed using CLSM. The red fluorescence represents the CD206 marker, green fluorescence represents the CD86 marker, and blue fluorescence represents the cell nuclei.

For WB, the cells were harvested and total proteins were extracted as previously described[13]. Briefly, the antibodies used for this assay were rabbit anti-CD206 (1:500; Abcam), rabbit anti-CD86 (1:500; Invitrogen), rabbit anti-iNOS (1:500; Invitrogen), rabbit anti-tubulin (1:1000; Cell Signaling Technology), and secondary goat anti-rabbit (1:3000; Beyotime Biotechnology). The protein levels of CD206, CD86, and iNOS were normalized to those of tubulin, which served as an internal reference.

To further evaluate the synergistic effect of KR-12 and TA on modulating the macrophage phenotype toward the M2 type, an immunofluorescence staining of CD86 and CD206 assay was used as mentioned above. There were four groups including control, HA/TA2, HA/KR2, and HA/TA2/KR2. The treatment of HA/TA2 and HA/KR2 represented the single action of TA and KR-12, respectively, and the treatment of HA/TA2/KR2 represented the synergistic action. All data were standardized with data from the control group.

For enzyme linked immunosorbent assay (ELISA), the supernatants in each well were harvested immediately, and then the protein levels of inflammatory cytokines including tumor necrosis factor α (TNF-α), interleukin 6 (IL-6) and interleukin 10 (IL-10) were measured using ELISA kits (Dakewe Biotech Co., China) according to the manufacturer's instructions.

To further evaluate the synergistic effect of KR-12 and TA on pro-inflammatory cytokines (TNF-α and IL-6), an ELISA was used as mentioned above. There were four groups including Control, HA/TA2, HA/KR2, and HA/TA2/KR2. The treatment of HA/TA2 and HA/KR2 represented the single action of TA and KR-12, respectively, and the treatment of HA/TA2/KR2 represented the synergistic action. All data were standardized with data from the control group.

12. Hemostasis assay

The hemostatic capacity of the cryogels was evaluated using the rat tail and liver injury model. For the rat tail injury model, Sprague Dawley rats were randomly and equally divided into four groups: a blank group (without any treatment), gauze group, HA/TA2/KR2 group, and alginate Ag group. The rats were anesthetized by injecting 10.0 wt% chloral hydrate (1 mL/300 g) intraperitoneally and fixed on the operation board. The tails were cut using surgical scissors of the same length and placed on pre-weighed filter paper. After cutting, tails were exposed to air for 10 s to ensure normal blood loss. The bleeding sites were covered in situ with gauze, HA/TA2/KR2 cryogel, and alginate Ag (Biatain@). The hemostatic time and weight of blood loss were recorded until no blood loss was observed.

For the rat liver injury model, rats were randomly and equally divided into four groups and anesthetized as described above. The livers of the rats were exposed by an abdominal incision, and a pre-weighed filter paper was placed beneath the liver. Bleeding was induced in the liver by using a needle to ensure normal blood loss. Subsequently, the bleeding sites were covered in situ with gauze, HA/TA2/KR2 cryogel, and alginate Ag. The hemostatic time and weight of blood loss were recorded until no blood loss was observed.

13. In vivo animal experiments

13.1. In vivo wound healing efficacy

All animal experiments were approved by the Laboratory Animal Welfare and Ethics Committee of the Army Medical University (AMUWEC20210410, AMUWEC20211786). A murine infected wound model was established according to our previous study with a little modification[14]. Briefly, BALB/c mice were anesthetized with 1% pentobarbital via intraperitoneal injection, and the dorsal hairs were shaved using a clipper. After being disinfected with 75% alcohol, two round wounds (diameter: 6mm) were carefully constructed on both sides of the dorsal skin, and the wounds were photographed as the initial data (day 0). Then, 10 uL of bacterial suspension of MDR-PA (1×10^8 CFU/mL) was uniformly dropped onto the wounds. After 12h, HA, HA/TA2 and HA/TA2/KR2 cryogels (diameter: 8mm) were attached to the wounds, respectively, and fixed with a 3M Tegaderm Film to prevent contraction as previously described[15]. Mice treated with gauze dressings served as control

group. On days 2, 4, 6 and 8 post-surgery, the appearances of wounds were photographed and the dressings were replaced. The unhealed wound areas in each group were measured using an Image J software and calculated as the following equation:

$$\text{Wound area (\%)} = A_n/A_0 \times 100\%$$

where A_n represents the wound areas of days 2, 4, 6 and 8, and A_0 represents the wound areas of day 0.

13.2. Hematoxylin and eosin (H&E) staining and histological analysis

On days 4 and 8 post-surgery, infected wound tissues (1 cm × 1 cm) in each group were harvested and fixed in 4% paraformaldehyde, followed by H&E staining. The H&E-stained sections were imaged using a full slide scanner (Olympus, Japan), and the length of the neo-epithelium, thickness of granulation tissue, and number of inflammatory cells were measured and analyzed using ImageJ software, according to our previous study[16]. On day 8 post-surgery, the mice were sacrificed, and the major organs, including the heart, liver, spleen, lungs, and kidneys, were harvested for H&E staining and histological analysis.

13.3. In vivo antibacterial activity

On day 4 post-surgery, infected wound tissues (1 cm × 1 cm) in each group were harvested and homogenized with 5 mL of physiological saline solution. Then, the number of bacterial colonies was counted using a standard plate counting method as described above. Furthermore, infected wound tissues were harvested and fixed in 4% formaldehyde, followed by dehydrated with a series of ethanol solution and sprayed with a gold layer. Finally, the samples were observed and analyzed using a SEM (Crossbeam 340, Zeiss, Germany).

13.4. In vivo antioxidation activity

On day 4 post-surgery, infected wound tissues (1 cm × 1 cm) in each group were harvested and sectioned by freezing microtome. Subsequently, the wound sections were stained using a ROS prober of dihydroethidium (DHE, Bjbalb, China) and a DAPI dye according to the manufacturer’s instructions. Finally, the staining wound sections were observed using a CLSM (Olympus, Tokyo, Japan). The red fluorescence represented ROS, and the blue fluorescence represented cell nuclei. The fluorescence intensity was quantified using ImageJ software.

13.5. Immunohistochemistry staining

Immunohistochemistry staining was performed as described in our previous study[17]. Briefly, after the wound sections were deparaffinized, rehydrated, and boiled in a 100 °C citrate buffer bath, they were incubated with primary antibodies overnight at 4 °C. The primary antibodies used were as follows: IL-6 (1:500, BioVision), TNF-α (1:500, Abcam), IL-10 (1:300, Invitrogen), PCNA (1:500, Cell Signaling Technology), and CD31 (1:200, Cell Signaling Technology). Then, the free primary antibodies were washed using PBS, and the sections were incubated with goat-anti-rabbit IgG antibody (Zhongshan Biology Company, China) or goat-anti-mouse IgG antibody (Zhongshan Biology Company, China) at 37 °C for 1 h. After washing with PBS, the wound sections were stained with 3,3'-diaminobenzidine tetrahydrochloride (DAB) solution and hematoxylin. Finally, the sections were imaged using a full-slide scanner (Olympus, Tokyo, Japan), and histological analysis was performed using ImageJ software.

13.6. In vivo immunofluorescence staining

Immunofluorescence staining was performed as previously described[13]. Briefly, the wound sections were successively deparaffinized, rehydrated and boiled in a 100°C citrate buffer bath. Then, the sections were incubated with primary antibodies including rabbit anti-iNOS antibody (1:200, Invitrogen) and rabbit anti-CD206 antibody (1:500, Abcam), respectively. After incubation overnight at 4 °C, the wound sections were washed with PBS, and incubated with Alexa Flour 488 goat anti-rabbit IgG (1:500, Beyotime Biotechnology, China) at 37 °C for 1 h. After co-staining with DAPI dye, the sections were imaged and analyzed using a CLSM (Olympus, Tokyo, Japan).

13.7. In vivo detection of releasing characteristic of KR-12

A murine wound model was established as mentioned above. Then, 10uL of different concentrations (0.2×10^8 CFU/mL, 1×10^8 CFU/mL, 5×10^8 CFU/mL) of bacterial suspensions of MDR-PA were uniformly dropped onto the wounds, respectively. The wounds inoculated with 10 uL of PBS served as a control group. After 12h, a piece of HA/TA2/KR2 cryogel (diameter: 8mm) was attached to the wounds in each group. The cryogel was harvested after application for 24 h, immersed in hyaluronidase solution (200 U/mL), and placed in a shaker

at 37 ° C. When the cryogel was completely degraded, the supernatant was collected to measure the concentration of KR-12 using an Ellman's assay as mentioned above. The release percentage of KR-12 was calculated using the equation:

$$\text{Release percentage (\%)} = (M - C \times V)/M \times 100\%$$

where C represents the concentration of KR-12, V represents the volume of hyaluronidase solution, and M represents the total mass of KR-12 in the cryogel.

14. Proteomic analysis

Label-free proteomic analysis was performed as previously described[18]. Briefly, the wound tissues of the control and HA/TA2/KR2 groups were harvested on day 4 postoperatively (n = 3) and immediately treated with liquid nitrogen. Total protein was extracted, and the protein concentration was measured using the Bradford method (Thermo Fisher Scientific, USA). The qualified peptide samples were digested with trypsin and desalinated using a C18 column (Waters, WAT054955, USA), and were then analyzed using a NanoLC-ESI-MS/MS system on the Thermo Scientific Orbitrap Exploris™480 platform (Thermo Fisher Scientific, USA) at Sichuan PANOMIX Biotechnology Co., Ltd. Proteins were identified and quantified using the Proteome Discoverer suite (version 2.4, Thermo Fisher Scientific, USA), and the spectral data were searched against the UniProtKB Mus musculus proteome database. The significant differentially expressed proteins were screened using a consistent standard (fold change ≥ 1.5 and P value < 0.05), and the KEGG pathway database (http://www.genome.jp/kegg/) and Gene Ontology database (http://www.geneontology.org/) were employed for further bioinformatics analyses. Western blotting (WB) was used for the verification of the proteomic analysis results as mentioned above. Briefly, the antibodies used for this assay were as follows: rabbit anti-NF-κb1 (1:1000, Cell Signaling Technology), rabbit anti-Stat6 (1:1000, ABclonal), rabbit anti-CD163 (1:1000, Abcam), rabbit anti-Arg1 (1:1000, Cell Signaling Technology), rabbit anti-Rhoa (1:1000, ABclonal), rabbit anti-Tubulin (1:1000, Cell Signaling Technology), and the secondary goat anti-rabbit (1:3000, Beyotime Biotechnology). Proteins of the same molecular weight were detected by eluting with a western blot fast stripping buffer (Shanghai Yamay Biomedical Technology Co., Ltd., China), and then incubated with primary antibodies again. The protein

levels of NF-κb1 (p105 and p50), Stat6, CD163, Arg1, and Rhoa were normalized to Tubulin which served as an internal reference.

15. Statistical analysis

The data in this study were expressed as the mean ± standard deviation (SD), and analyzed using the unpaired t-test and one-way ANOVA with Turkey test by GraphPad Prism 8.0 software (GraphPad Software Corporation, USA). Data plotting was performed using GraphPad Prism 8.0 and Origin 2021 software (Origin Lab Corporation, USA). All experiments were conducted with a minimum of three in each group. Statistical significance was set as $P < 0.05$ (“*”) and $P < 0.01$ (“**”).

Reference

1. Turell, L.; Zeida, A.; Trujillo, M., Mechanisms and consequences of protein cysteine oxidation: the role of the initial short-lived intermediates. *Essays In Biochemistry* **2020**, *64* (1), 55-66.

2. Tang, S.; Chi, K.; Xu, H.; Yong, Q.; Yang, J.; Catchmark, J. M., A covalently cross-linked hyaluronic acid/bacterial cellulose composite hydrogel for potential biological applications. *Carbohydrate Polymers* **2021,** *252*, 117123.

3. Lee, C. H.; Song, S. Y.; Chung, Y. J.; Choi, E. K.; Jang, J.; Lee, D. H.; Kim, H. D.; Kim, D. U.; Park, C. B., Light-stimulated carbon dot hydrogel: targeting and clearing infectious bacteria in vivo. *ACS applied bio materials* **2022,** *5* (2), 761-770.

4. Zhang, J.; Wu, M. M.; Peng, P.; Liu, J. Q.; Lu, J.; Qian, S. X.; Feng, J., "Self-defensive" antifouling zwitterionic hydrogel coatings on polymeric substrates. *ACS Applied Materials & Interfaces* **2022,** *14* (50), 56097-56109.

5. Yuwen, L. H.; Qiu, Q.; Xiu, W. J.; Yang, K. L.; Li, Y. Q.; Xiao, H.; Yang, W. J.; Yang, D. L.; Wang, L. H., Hyaluronidase-responsive phototheranostic nanoagents for fluorescence imaging and photothermal/photodynamic therapy of methicillin-resistant Staphylococcus aureus infections. *Biomaterials Science* **2021,** *9* (12), 4484-4495.

6. Patel, A.; Goswami, S.; Hazarika, G.; Sivaprakasam, S.; Bhattacharjee, S.; Manna, D., Sulfonium-cross-linked hyaluronic acid-based self-healing hydrogel: stimuli-responsive drug

carrier with inherent antibacterial activity to counteract antibiotic-resistant bacteria. *Advanced Healthcare Materials* **2023,** e2302790.

7. Ahmadian, Z.; Correia, A.; Hasany, M.; Figueiredo, P.; Dobakhti, F.; Eskandari, M. R.; Hosseini, S. H.; Abiri, R.; Khorshid, S.; Hirvonen, J.; Santos, H. A.; Shahbazi, M. A., A hydrogen-bonded extracellular matrix-mimicking bactericidal hydrogel with radical scavenging and hemostatic function for pH-responsive wound healing acceleration. *Advanced Healthcare Materials* **2021,** *10* (3), e2001122.

8. Song, D. W.; Kim, S. H.; Kim, H. H.; Lee, K. H.; Ki, C. S.; Park, Y. H., Multi-biofunction of antimicrobial peptide-immobilized silk fibroin nanofiber membrane: implications for wound healing. *Acta Biomaterialia* **2016,** *39*, 146-155.

9. Zou, C. Y.; Lei, X. X.; Hu, J. J.; Jiang, Y. L.; Li, Q. J.; Song, Y. T.; Zhang, Q. Y.; Li-Ling, J.; Xie, H. Q., Multi-crosslinking hydrogels with robust bio-adhesion and pro-coagulant activity for first-aid hemostasis and infected wound healing. *Bioactive Materials* **2022,** *16*, 388-402.

10. Feng, Z.; Su, Q.; Zhang, C.; Huang, P.; Song, H.; Dong, A.; Kong, D.; Wang, W., Bioinspired nanofibrous glycopeptide hydrogel dressing for accelerating wound healing: a cytokine-free, M2-type macrophage polarization approach. *Advanced Functional Materials* **2020,** *30* (52), 2006454.

11. Xu, S. B.; Chang, L. N.; Hu, Y. A.; Zhao, X. J.; Huang, S. C.; Chen, Z. H.; Ren, X. L.; Mei, X. F., Tea polyphenol modified, photothermal responsive and ROS generative black phosphorus quantum dots as nanoplatforms for promoting MRSA infected wounds healing in diabetic rats. *Journal of Nanobiotechnology* **2022,** *20* (1), 192.

12. Zhou, L. S.; Zhou, L. M.; Wei, C. X.; Guo, R., A bioactive dextran-based hydrogel promote the healing of infected wounds via antibacterial and immunomodulatory. *Carbohydrate Polymers* **2022,** *291*, 119558.

13. Xu, N.; Gao, Y.; Li, Z.; Chen, Y.; Liu, M.; Jia, J.; Zeng, R.; Luo, G.; Li, J.; Yu, Y., Immunoregulatory hydrogel decorated with Tannic acid/Ferric ion accelerates diabetic wound healing via regulating Macrophage polarization. *Chemical Engineering Journal* **2023,** *466*, 143173.

14. Liu, M.; Liu, T.; Chen, X.; Yang, J.; Deng, J.; He, W.; Zhang, X.; Lei, Q.; Hu, X.; Luo, G.; Wu, J., Nano-silver-incorporated biomimetic polydopamine coating on a thermoplastic polyurethane porous nanocomposite as an efficient antibacterial wound dressing. *Journal of Nanobiotechnology* **2018,** *16* (1), 89.

15. Yao, Z.; Huang, Y.; Luo, G.; Wu, J.; He, W., A biological membrane-based novel excisional wound-splinting model in mice (with video). *Burns & Trauma* **2014,** *2* (4), 196-200.

16. Liu, M.; Luo, G.; Wang, Y.; He, W.; Liu, T.; Zhou, D.; Hu, X.; Xing, M.; Wu, J., Optimization and integration of nanosilver on polycaprolactone nanofibrous mesh for bacterial inhibition and wound healing in vitro and in vivo. *International Journal of Nanomedicine* **2017,** *12*, 6827-6840.

17. Liu, M.; Liu, T.; Zhang, X.; Jian, Z.; Xia, H.; Yang, J.; Hu, X.; Xing, M.; Luo, G.; Wu, J., Fabrication of KR-12 peptide-containing hyaluronic acid immobilized fibrous eggshell membrane effectively kills multi-drug-resistant bacteria, promotes angiogenesis and accelerates re-epithelialization. *International Journal of Nanomedicine* **2019,** *14*, 3345-3360.

18. Wang, W.; Luo, J.; Sheng, W.; Xue, J.; Li, M.; Ji, J.; Liu, P.; Zhang, X.; Cao, J.; Zhang, S., Proteomic profiling of radiation-induced skin fibrosis in rats: targeting the ubiquitin-proteasome system. *International Journal of Radiation Oncology, Biology, Physics* **2016,** *95* (2), 751-60.

Supporting images and tables

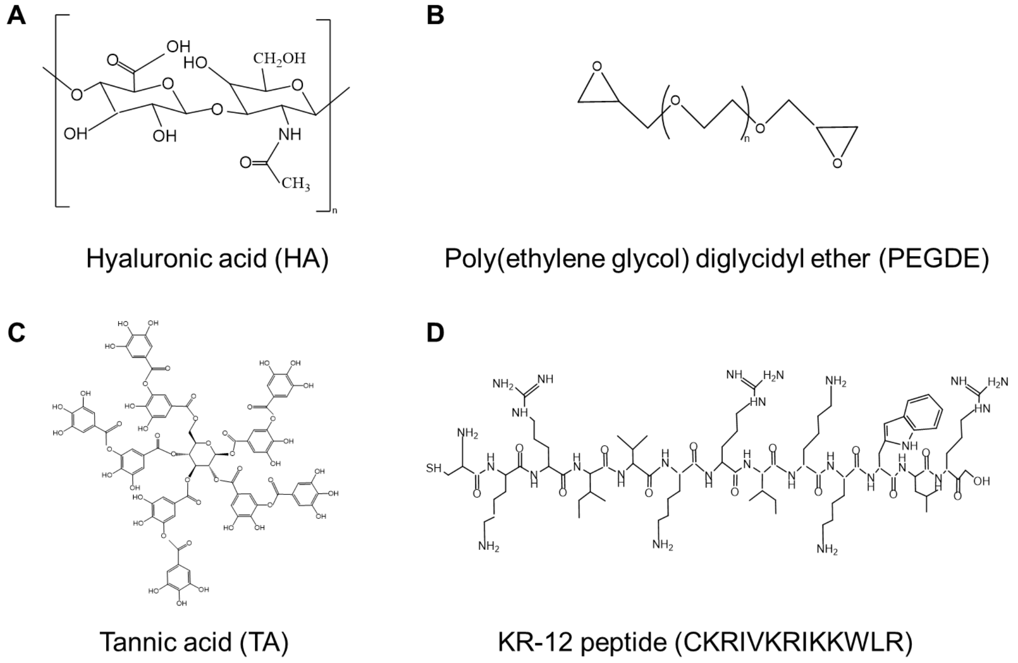

Figure S1. Molecular structures. (**A**) Hyaluronic acid. (**B**) Poly(ethylene glycol) diglycidyl ether. (**C**) Tannic acid. (**D**) KR-12 peptide.

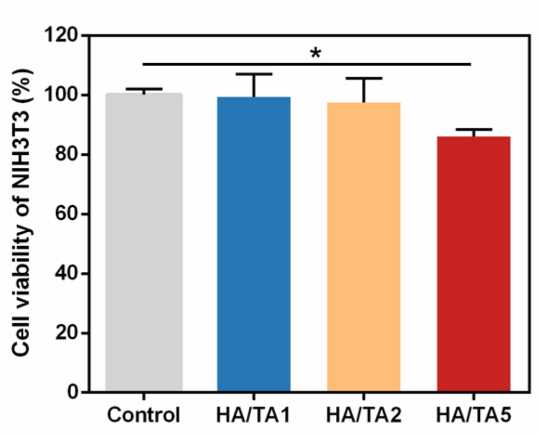

Figure S2. Cytocompatibility of HA/TA cryogels. The cytocompatibility of HA/TA cryogels containing 0.1%, 0.2% and 0.5% (w/v) TA (denoted as HA/TA1, HA/TA2 and HA/TA5, respectively) were tested by cell viability of NIH3T3 cells (n = 4). P < 0.05 ("*") and P < 0.01 ("**").

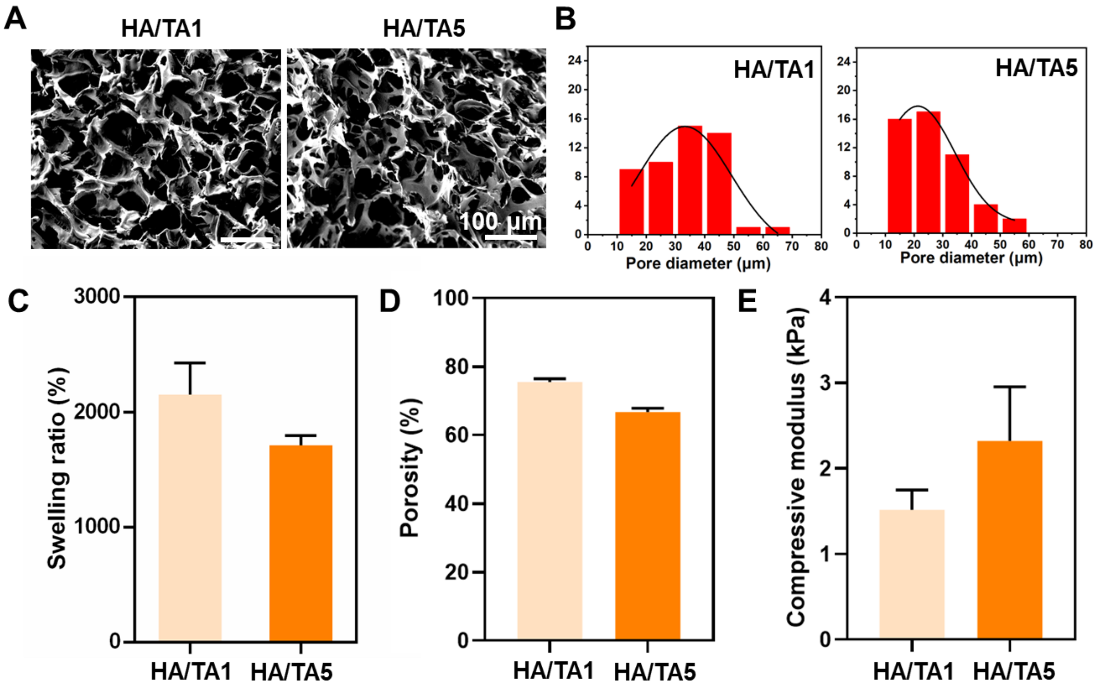

Figure S3. Physical properties of the HA/TA cryogels related to the concentration of TA. Morphology and pore diameter (A-B), swelling ratio (C), porosity (D) and compressive modulus (E) of HA/TA cryogels vs the concentration of TA.

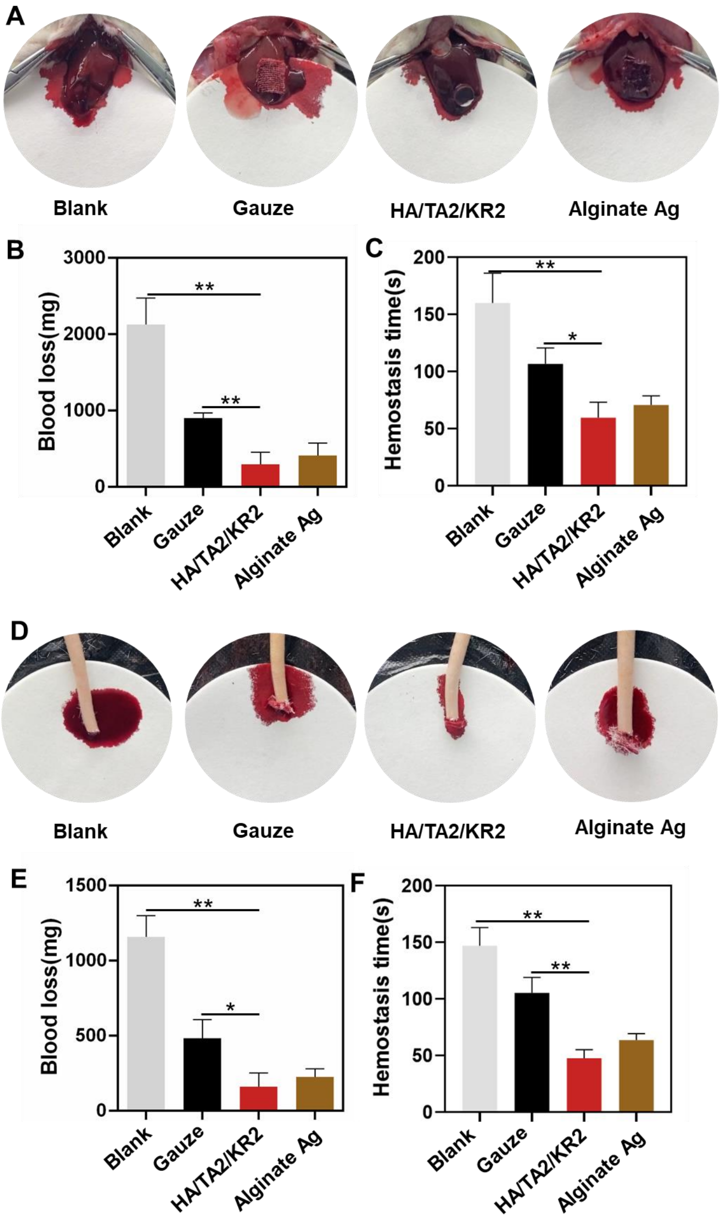

Figure S4. Hemostatic performance. (**A**) Images of liver hemostasis in blank, gauze, HA/TA2/KR2 and alginate Ag groups. (**B-C**) Liver blood loss and hemostasis time of various groups (n = 3), corresponding to (A). (**D**) Images of tail hemostasis in blank, gauze, HA/TA2/KR2 and alginate Ag groups. (**E-F**) Tail blood loss and hemostasis time of various groups (n = 3), corresponding to (D). $P < 0.05$ (“*”) and $P < 0.01$ (“**”).

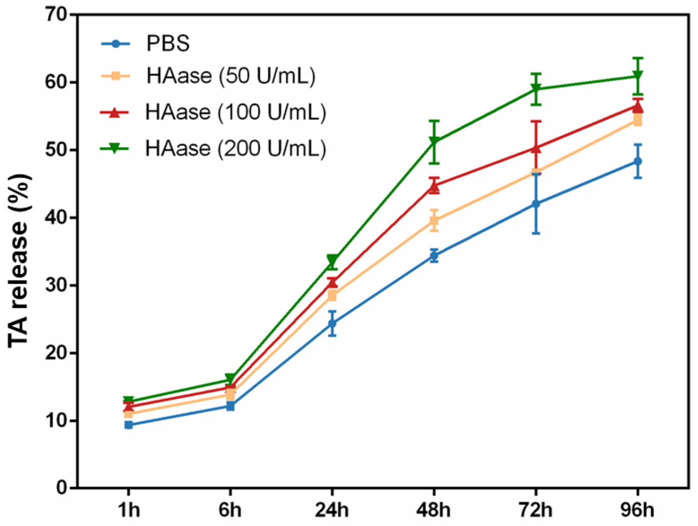

Figure S5. Release behavior of TA induced by hyaluronidase (HAase). Release percentage of TA from HA/TA2/KR2 cryogel mediated by HAase with various concentrations (0, 50, 100, and 200 U/mL) for 96 h (n = 3).

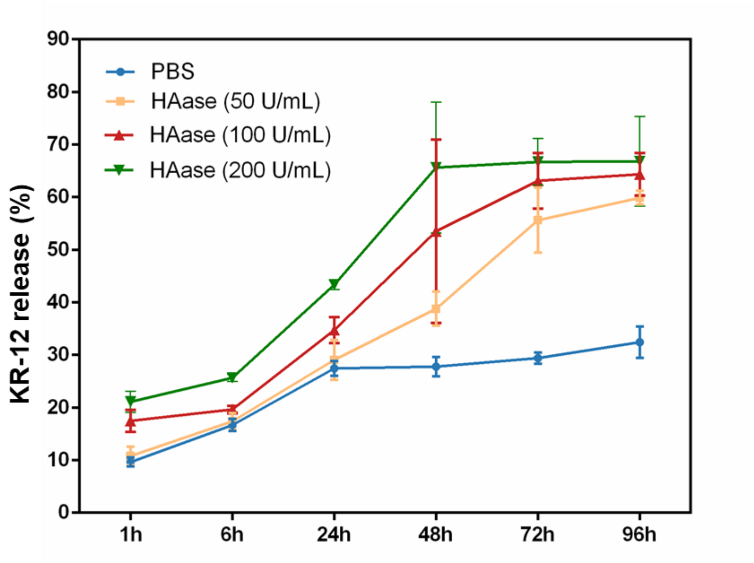

Figure S6. Release behavior of KR-12 induced by HAase. Release percentage of KR-12 from HA/TA2/KR2 mediated by HAase with various concentrations (0, 50, 100, and 200 U/mL) for 96 h (n = 3).

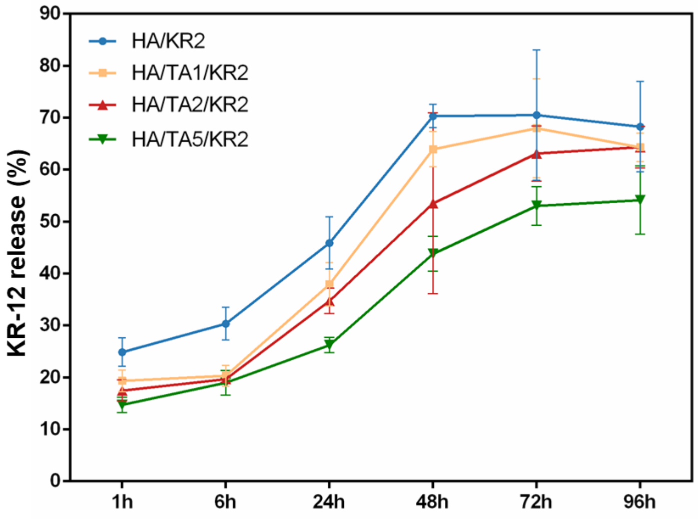

Figure S7. Release behavior of KR-12 under different concentrations of TA binding. Release percentage of KR-12 was negatively correlated with the concentration of TA in cryogel, where the concentration of TA varied from 0 to 0.5% (w/v) and HAase was at the concentration of 100 U/mL (n = 3).

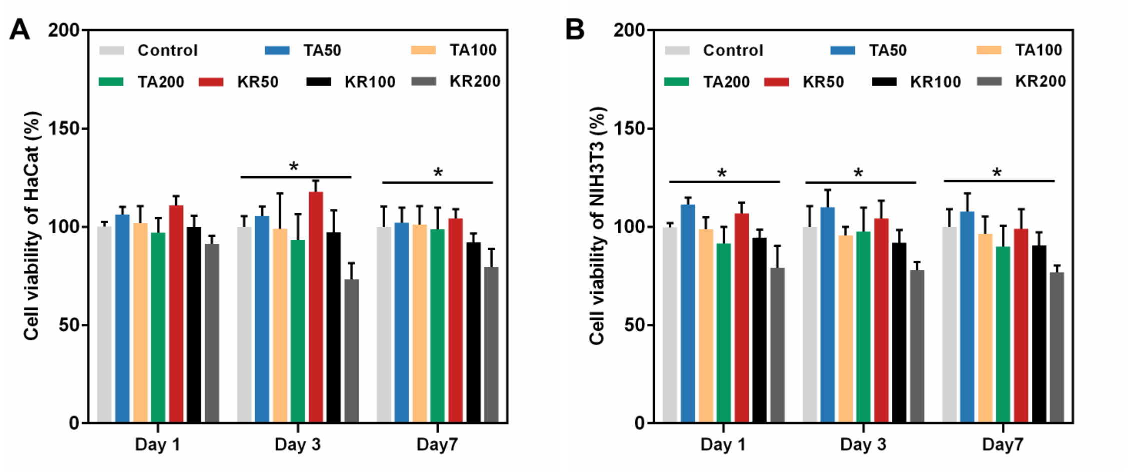

Figure S8. Cytotoxicity of free TA and KR-12. (A) Cell viability of HaCat cells treated with free TA (50, 100, and 200 μg) and KR-12 (50, 100, and 200 μg) (n = 4). (B) Cell viability of NIH3T3 cells treated with free TA (50, 100, and 200 μg) and KR-12 (50, 100, and 200 μg) (n = 4). $P < 0.05$ ("*") and $P < 0.01$ ("**").

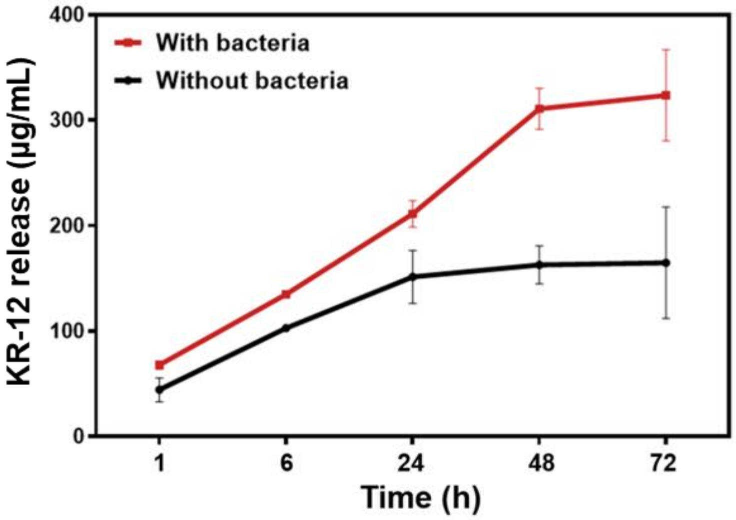

Figure S9. Release behavior of KR-12 in different environments with bacteria or without bacteria (n = 3).

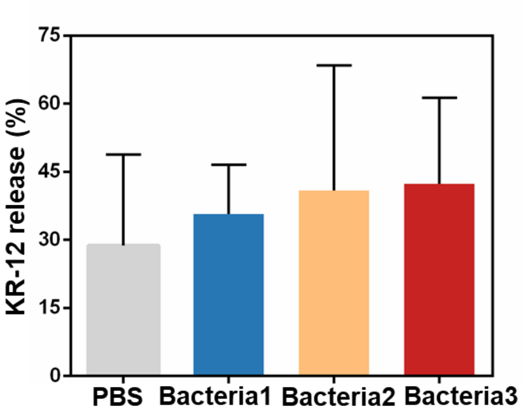

Figure S10. In vivo release behavior of KR-12 on wounds infected with various initial concentrations of bacteria. The release percentage of KR-12 on wounds infected with 10 μL of bacteria at different concentrations (0, 0.2×10^8, 1×10^8, and 5×10^8 CFU/mL, denoted as PBS, Bacteria1, Bacteria2, Bacteria3) for 24 h (n = 3).

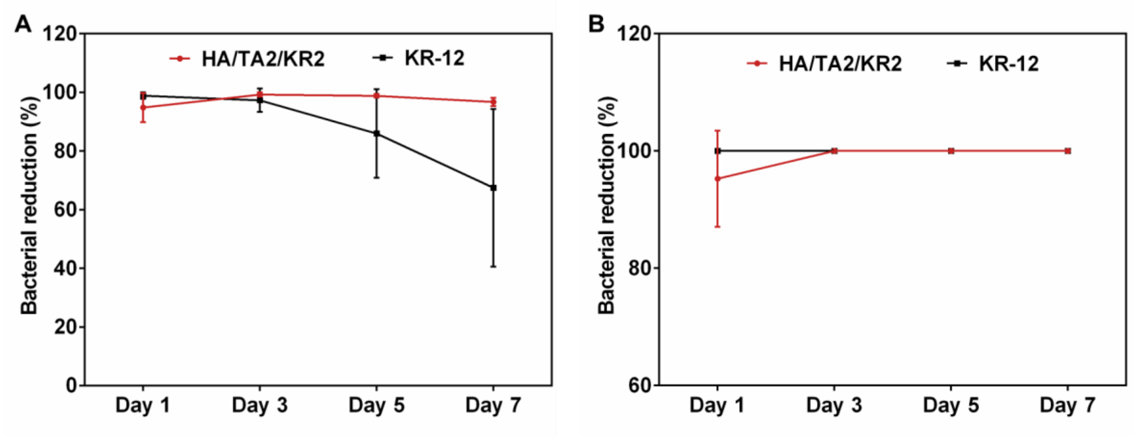

Figure S11. Long-term antibacterial property. (A) Anti-MRSA curve of HA/TA2/KR2 cryogel and free KR-12 (equivalent mass to the KR-12 in cryogel) for 7 days (n = 3). (B) Anti-MDR-PA curve of HA/TA2/KR2 and free KR-12 for 7 days (n = 3).

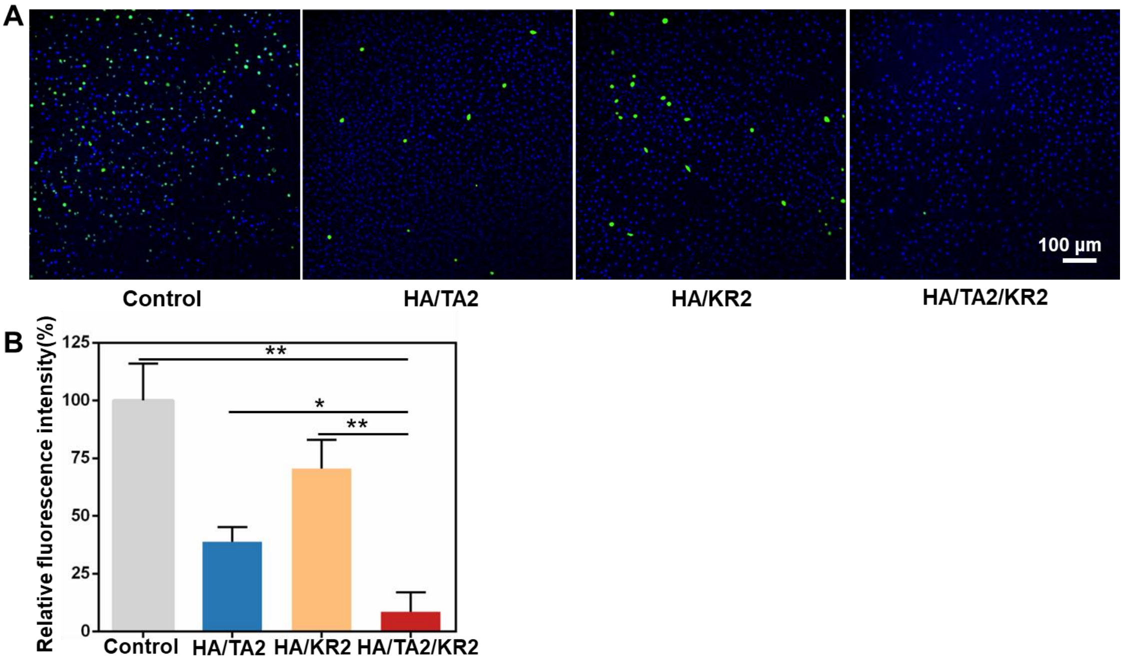

Figure S12. Synergistic ROS scavenging effect of TA and KR-12. (A) Fluorescence images of ROS treated with control, HA/TA2, HA/KR2, and HA/TA2/KR2. (B) Quantitative analysis of relative ROS expression corresponding to (A) (n = 3). P < 0.05 ("*") and P < 0.01 ("**").

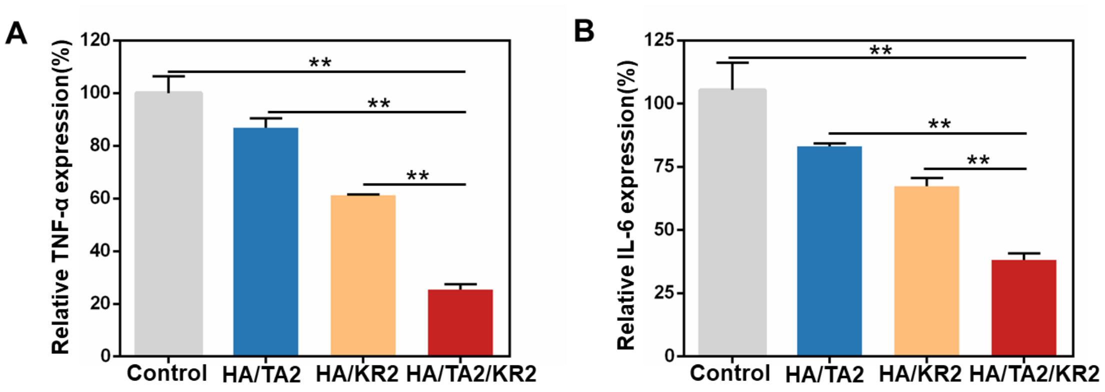

Figure S13. Synergistic anti-inflammatory effect of TA and KR-12. (A) The relative expression of TNF-α measured using ELISA in control, HA/TA2, HA/KR2 and HA/TA2/KR2 groups (n = 3). (B) The relative expression of IL-6 measured using ELISA in control, HA/TA2, HA/KR2 and HA/TA2/KR2 groups (n = 3). The results showed that coexist of TA and KR-12 exhibited the strongest anti-inflammatory effect (n = 3). $P < 0.05$ ("*") and $P < 0.01$ ("**").

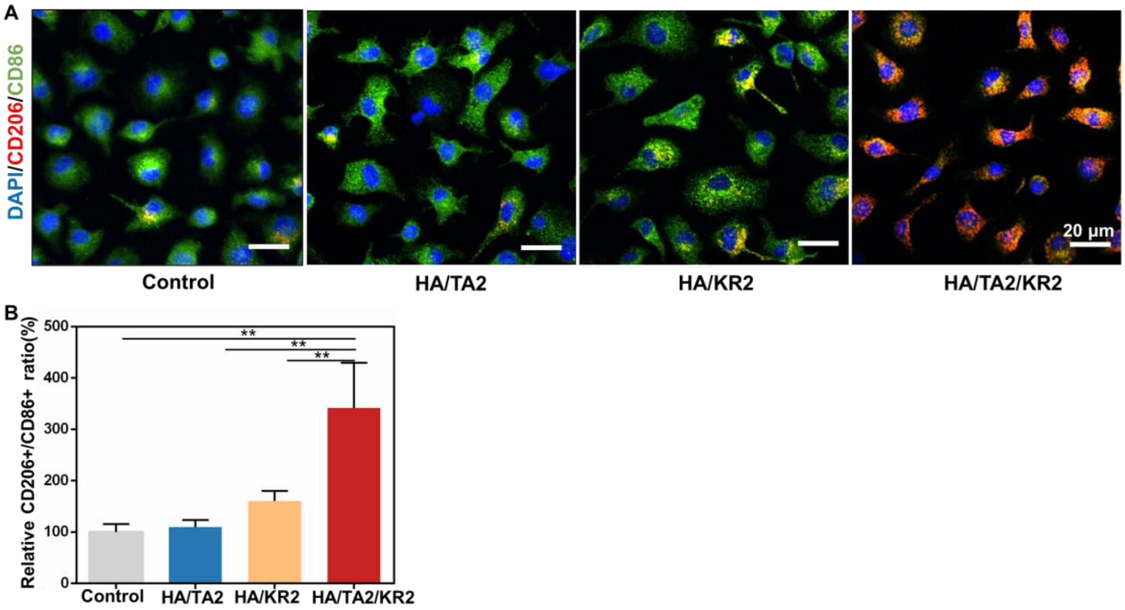

Figure S14. Synergistic effect of TA and KR-12 on polarization of macrophages towards the M2 type. (**A**) Immunofluorescence images of BMDMs from the control, HA/TA2, HA/KR2, and HA/TA2/KR2 groups. (**B**) Ratios of $CD206^+/CD86^+$ expression relative to control in the HA/TA2, HA/KR2, and HA/TA2/KR2 groups (n = 3). $P < 0.05$ ("*") and $P < 0.01$ ("**").

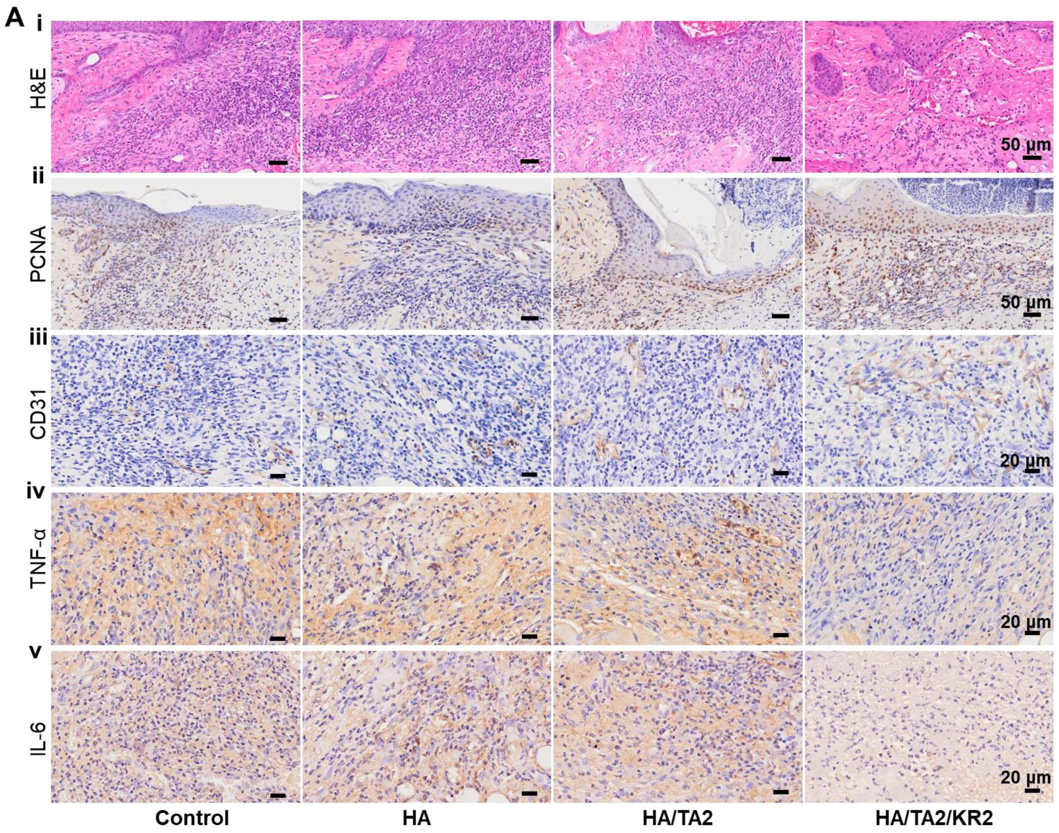

Figure S15. Inflammatory cells infiltration, pro-healing markers and inflammatory cytokines expression in wound tissues. (**A**) H&E stains (Ai), and immunohistochemical stains of PCNA (Aii), CD31 (Aiii), TNF-α (Aiv), and IL-6 (Av) of wound tissues from control, HA, HA/TA2 and HA/TA2/KR2 groups on the 8th day.

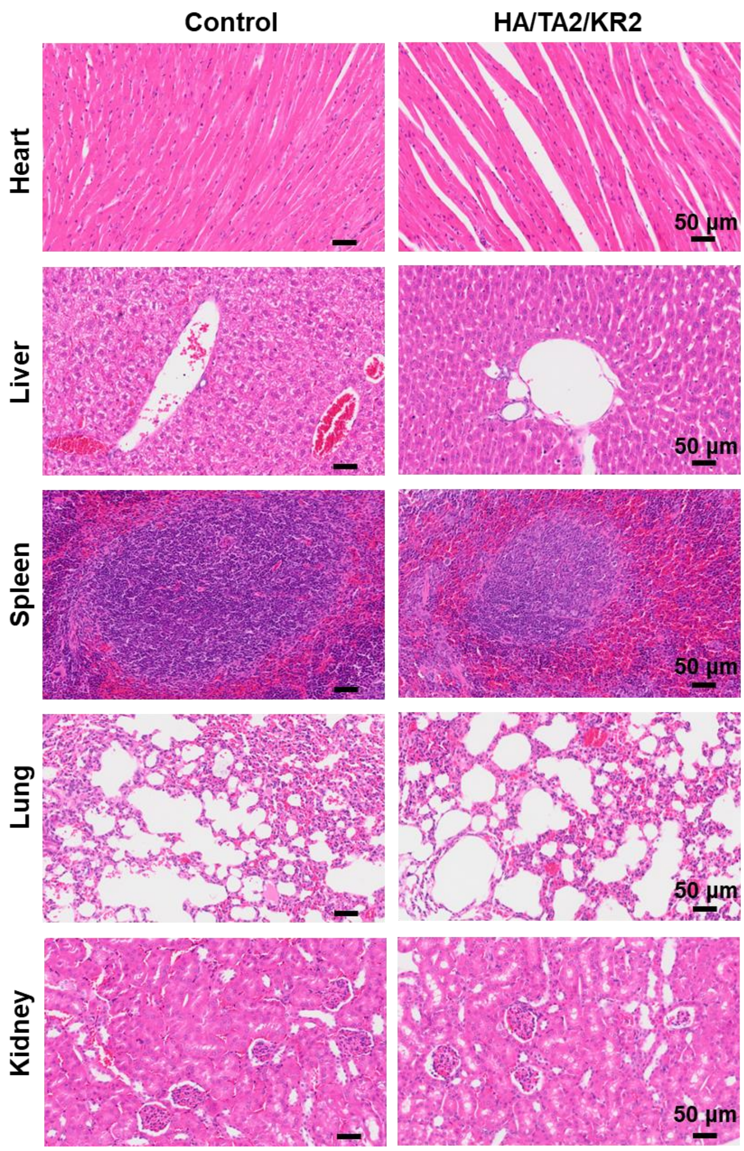

Figure S16. Histology stains of visceral organs. H&E stains of heart, liver, spleen, lung and kidney from the control and HA/TA2/KR2 groups on the 8th day.

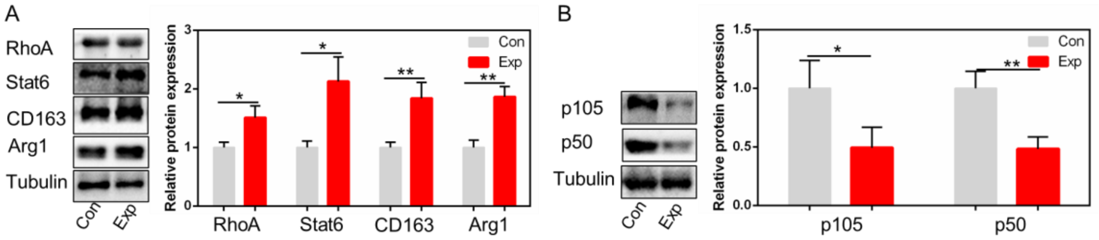

Figure S17. Protein expression detected using WB. (A) WB images and quantitative analysis of proteins in control and experimental (HA/TA2/KR2) groups, where marked proteins including RhoA, Stat6, CD163, and Arg1(n = 3). (B) WB images and quantitative analysis of proteins NF-κb1 (p105 and p50) in control and experimental groups (n = 3). $P < 0.05$ ("*") and $P < 0.01$ ("**").
